# Hadamard-Based Recursive Aperture Decoded Ultrasound Imaging (READI) With Estimated Motion-Compensated Compounding (EMC^2^) Using Top-Orthogonal-to-Bottom-Electrode (TOBE) Arrays

**DOI:** 10.1109/TUSON.2026.3704971

**Published:** 2026-08

**Authors:** Tyler Keith Henry, Darren Dahunsi, Randy Palamar, Negar Majidi, Ying Wan, Mohammad Rahim Sobhani, Afshin Kashani Ilkhechi, Roger Zemp

**Affiliations:** Department of Electrical and Computer Engineering, University of Alberta, Edmonton, AB T6G 1H9, Canada

**Keywords:** Flow estimation, Hadamard aperture encoding, motion compensation, synthetic transmit aperture (STA)

## Abstract

Hadamard matrix-based aperture encoding is a method for producing synthetic aperture datasets with high signal-to-noise ratios (SNRs). Recently, the pulse inversion capabilities of bias-sensitive top-orthogonal-to-bottom-electrode (TOBE) arrays have driven the development of multiple Hadamard-based sequences. These sequences produce high-quality static images but are sensitive to motion. This work introduces recursive aperture decoded ultrasound imaging (READI) and estimated motion-compensated compounding (EMC^2^), which look to reduce this sensitivity. READI is a novel decoding and beamforming technique for Hadamard aperture-encoded sequences that produces multiple low-resolution images from subsets of the full sequence. These READI images are less affected by motion and sum to form the complete high-resolution image. EMC^2^ describes the process of comparing these low-resolution images to estimate the underlying motion, then warping them to align before compounding. This produces a high-resolution image that is resilient to motion. READI with EMC^2^ applied to the TOBE-based fast orthogonal row–column electronic scanning (FORCES) sequence. It is shown to fully restore images corrupted by probe motion and to recover tissue speckle and boundaries in images of a beating heart phantom. READI low-resolution images by themselves are demonstrated to be a marked improvement over a sparse Hadamard scheme with the same transmit count, and are able to recover blood speckle at a flow rate of 42 cm/s.

## Graphical Abstract

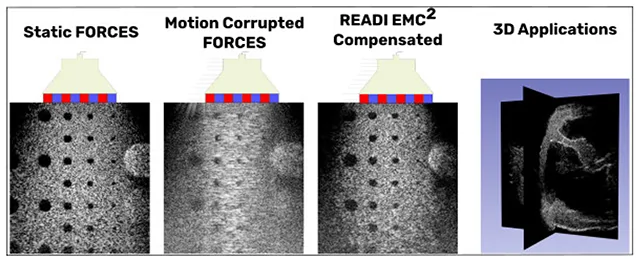

## I. Introduction

Synthetic transmit aperture (STA) ultrasound imaging is a well-established alternative to traditional scan-line imaging that offers global transmit and receive focusing [1]. STA combines a series of unfocused pulses from small subapertures into a large “synthetic” aperture spanning the entire array. The diverging transmissions maximize image width, while frame rate can be increased using sparse transmit schemes [2], [3].

Fast orthogonal row-column electronic scanning (FORCES) [4], [5] is a recently developed 2-D STA imaging technique that utilizes Hadamard aperture encoding with bias-sensitive top-orthogonal-to-bottom-electrode (TOBE) arrays [6] to achieve the same synthetic aperture as STA while transmitting with the complete array on each event. This maximizes the total energy transmitted into the volume, boosting signal-to-noise ratio (SNR) and penetration of all signals.

Motion in the imaged volume during an STA sequence can prevent the transmits from coherently compounding and lead to significant image blur [1], [7]. This effect is exacerbated by FORCES, as motion also prevents proper decoding of the aperture. Sparse STA transmit schemes can increase the frame rate and reduce motion artifacts; this has been applied to FORCES [8] but comes at the cost of SNR, the value FORCES is designed to maximize.

This work introduces recursive aperture decoded ultrasound Imaging (READI), a new decoding and beamforming technique for Hadamard aperture-encoded sequences such as FORCES. READI separates the encoded dataset into sequential groups of transmit events, partially decodes them using a smaller Hadamard matrix, and then beamforms each group into a low-resolution image. These READI images are individually less corrupted by motion and, when combined, form the complete image at its full resolution and SNR. The underlying motion can be determined by comparing these READI images and reversed by warping them. They can then form the complete image with improved motion resilience. We refer to this new process as estimated motion-compensated compounding (EMC^2^). These methods were developed for and tested on the FORCES sequence, but in principle can be applied to any Hadamard aperture-encoded sequence.

This article includes cross-plane/3-D renderings and videos of a human-sized beating-heart phantom with realistic motion and heart rates. The results indicate the potential for cross-plane imaging of the entire human heart using bias-switchable TOBE arrays, where most previous row-column arrays (RCAs) have been limited to 3-D imaging below the shadow of the aperture [5]. It demonstrates robustness to motions encountered in future clinical scenarios and offers a combination of image quality, frame rate, and field of view not previously achieved with RCA methods.

Five experiments were conducted to evaluate the proposed methods. First, a FORCES acquisition was simulated using Field II [9], [10] to validate that compounded READI images perfectly reproduce the FORCES image. The resolution of READI images of various group sizes is also compared, along with the relative effects of motion. Second, a static anechoic cyst phantom was imaged with READI and compared with ultrafast FORCES (uFORCES) [8], the aforementioned sparse version of FORCES. Then, the same phantom was imaged while the probe was in motion to determine if EMC^2^ can recover the static image. Next, a beating heart phantom was imaged in motion and compensated with EMC^2^ to evaluate performance on nonuniform tissue motion. Finally, a flow phantom was imaged to determine if READI images can resolve blood flow and speckle.

## II. Background

This section will provide a high-level overview of TOBE arrays and their unique capabilities, followed by a detailed review of FORCES. It will cover how FORCES improves upon STA with Hadamard encoding, how the dataset is beamformed, and the unique benefits of FORCES imaging with TOBE arrays, including multiplane imaging. The equations introduced in Section II-B are necessary for the derivation of READI in Section III.

### A. TOBE Arrays

**Fig. 1.**
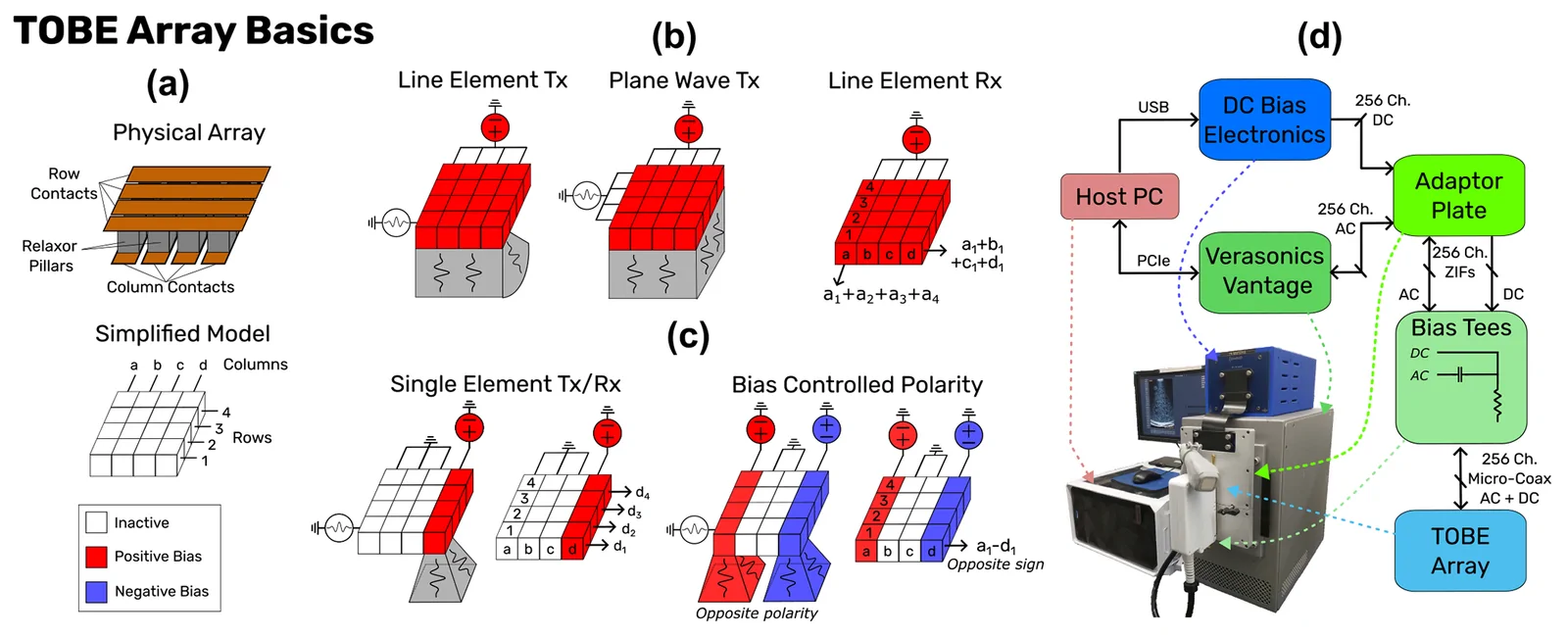
Operation of a relaxor-based TOBE array. (a) Physical layout of elements and channels. (b) RCA functionality achieved by biasing all elements uniformly. (c) Unique TOBE array functionality enabled by bias control. (d) Hardware block diagram for a TOBE array and ultrasound system.

TOBE arrays [6] are a refinement of RCA [11], [12], [13] that allow for individual element addressing while retaining the same channel count and geometry. In a standard RCA, elements are organized into an *N* × *M* grid, where each element is connected to one row and one column contact. When transmitting or receiving along a contact, all connected elements transmit simultaneously and contribute equally to the received signal. TOBE arrays eliminate this coupling by using electrostrictive relaxor materials, which exhibit a piezoelectric response that is linearly proportional to an external DC bias [14], [15]. In the absence of this bias, the elements will not transmit or receive signals, while two equal and oppositely biased elements will have inverted responses. Fig. 1 highlights the construction and biasing of a TOBE array, along with a complete block diagram of the imaging system with custom DC bias electronics. This work uses relaxor-based TOBE arrays; however, they can also be constructed from capacitive micromachined ultrasonic transducers (CMUTs) [6].

If all elements are biased with a fixed voltage, then TOBE arrays act identically to traditional RCAs [Fig. 1(b)] and are capable of performing the same imaging sequences [5]. In addition, their bias-modulation capabilities [Fig. 1(c)] make them well-suited to new methods such as Hadamard aperture encoding. This spatial encoding requires some elements to transmit inverted pulses, which are difficult to implement on many clinical systems without modification [16], [17]. TOBE arrays can instead linearly invert the pulse by applying a negative bias to the transmitting element(s) as demonstrated in Fig. 1(c). Multiple TOBE imaging sequences have been implemented with this encoding, such as FORCES [4] and Hadamard encoded row-column ultrasonic expansive scanning (HERCULES) [18].

TOBE arrays enable individual-element addressing with a significantly reduced channel count compared to fully wired matrix arrays. Current commercially available matrix arrays are typically limited to 32 × 32 elements with 1024 channels [19], [20], and often require additional multiplexing [21]. In comparison, a 128 × 128 element TOBE array requires only 256 channels. Matrix probes with embedded microbeamformers, such as Philips’ xMatrix [22], [23], can reduce the channel count but require complex in-probe electronics [24] that are difficult to scale to larger arrays. Sparse 2-D arrays have also been proposed to reduce channel count [25], [26], but suffer from low SNR and poor image quality [27].

### B. FORCES Imaging

#### 1) The Multistatic Dataset and Hadamard Aperture Encoding:

The goal of FORCES is to produce a “multistatic” dataset for a 1-D linear array, a set of signals representing every combination of transmit and receive elements [28]. This dataset can be defined as an *N* × *M* matrix of signals

(1) $$S(t)\in {\mathbb{R}}^{N\times M}\;S(t)=\left[\begin{array}{ccc} s_{1,1}(t) & \cdots & s_{1,M}(t)\\ \vdots & \ddots & \vdots \\ s_{N,1}(t) & \cdots & s_{N,M}(t) \end{array}\right]$$

where *N* and *M* are the number of transmit and receive elements, respectively, with *s_ij_*(*t*) representing the signal received on element *j*, transmitted by element *i*.

**Fig. 2.**
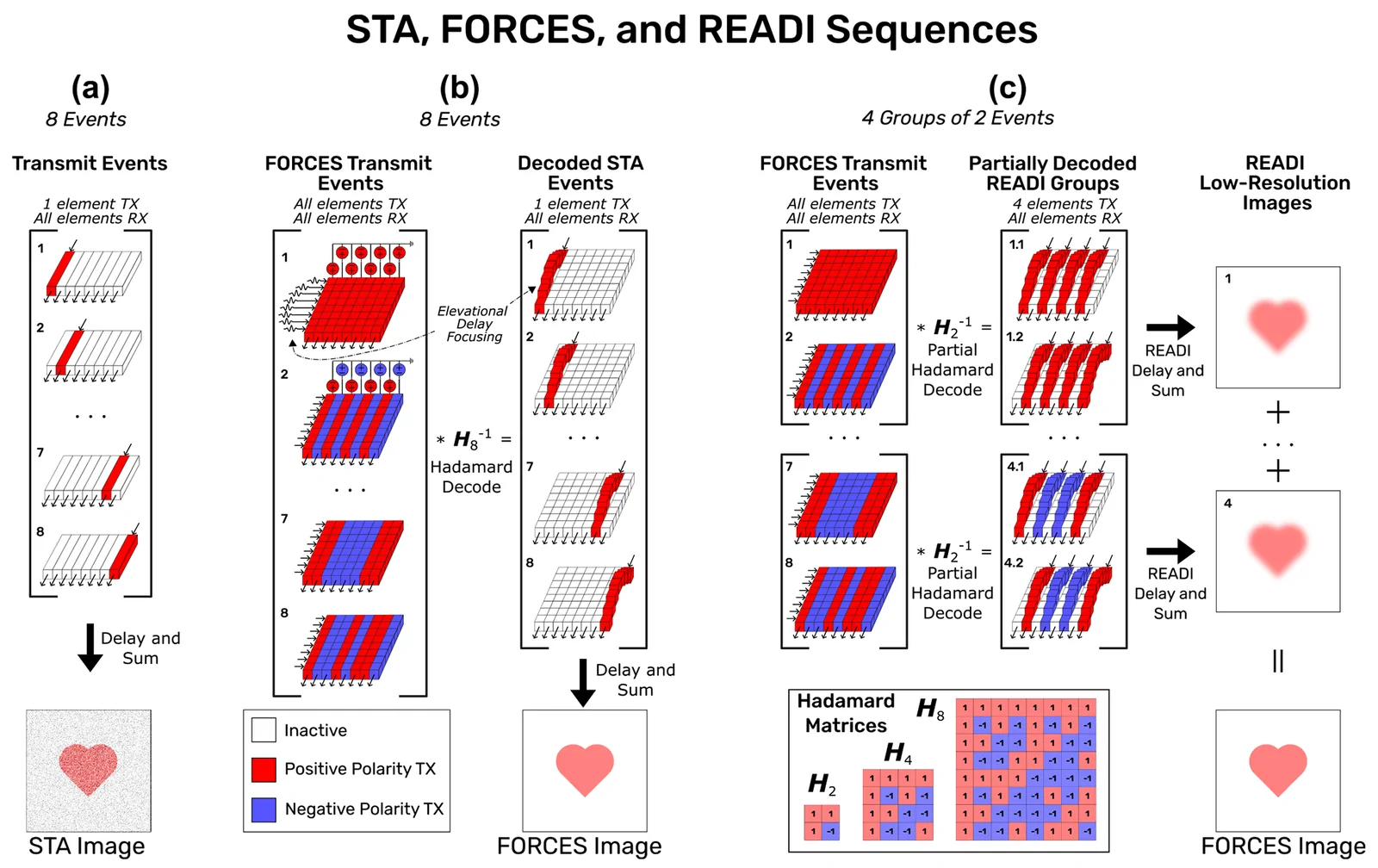
Comparison between eight transmit (a) STA, (b) FORCES, and (c) READI sequences. The biasing for each FORCES transmit event is taken from the rows of the ***H*_8_** matrix at the bottom. READI breaks the FORCES sequence into groups of 2, and partially decodes them using $H_{2}^{-1}$. Each partially decoded group is beamformed into a READI low-resolution image, which is then summed to form the FORCES image. The FORCES transmit events also include elevational delay focusing, which is annotated in the first event of B and represented by the arcing columns in the decoded events.

In FORCES, we receive on all channels along the lateral direction, like with standard RCAs [Fig. 1(b)]. This provides a unique signal for all *M* receive elements, while the contributions of the *N* transmitting elements are combined together. Standard STA imaging separates these contributions by firing one element per event. After *N* events, the full multistatic dataset has been recorded and is directly beamformed. This is illustrated in Fig. 2(a) with an eight-element linear array. The remainder of this analysis focuses on how FORCES separates these transmit contributions; for simplicity, we consider only a single receive channel (*M* = 1).

FORCES transmits on all lateral channels each event, with apodizations taken from a Hadamard matrix [Fig. 2(b)] [29]. This is a square matrix consisting of only +1 and −1 elements, with the property that all rows and columns are mutually orthogonal. Hadamard matrices can be constructed with a recursive formula

(2) $$H_{2}=\left(\begin{array}{c} +1,+1\\ +1,-1 \end{array}\right)\;H_{2^{a+b}}=H_{2^{a}}⊗H_{2^{b}},\;a,b\in \mathbb{N}.$$

Note that this construction always yields a diagonally symmetric matrix with ***H*** = ***H***^*T*^. It is an open conjecture that a Hadamard matrix exists for all multiples of four, while the construction in (2) is limited to powers of two.

If a Hadamard apodization pattern is applied to the aperture for each event, the resulting dataset **G**(t) can be thought of as a linear combination of the multistatic dataset **S**(t) weighted by the Hadamard matrix

(3) $${\text{g}}_{e}(t)=\sum_{i=1}^{N}h_{ei}^{\left(N\right)}s_{i}(t)\;G(t)=H_{N}S(t)$$

(4) $$S(t)=H_{N}^{-1}G(t)$$

where for each event *e*, the received signal g_*e*_(*t*) is a linear combination of the signals from each transmit element *s_i_*(*t*) weighted by the corresponding element of the Hadamard matrix $h_{\mathrm{ei}}^{\left(N\right)}$. Thus, as Hadamard matrices are invertible, the original multistatic dataset can be recovered by applying the inverse of the Hadamard matrix ($H_{N}^{-1}$) to the received dataset.

Fig. 2(b) demonstrates the FORCES sequence. The physical transmit events (left column) are biased according to the Hadamard matrix and include an elevational delay profile. After multiplying the eight received signals by the inverse matrix ($H_{8}^{-1}$), a dataset with the same lateral transmit geometry as STA is recovered (right column). The dataset is beamformed in the same way as STA, but produces a higher quality image.

#### 2) DAS Beamforming:

Once the multistatic dataset **S**(t) is recovered, it can be beamformed to produce an image. The most common method of beamforming is known as delay-and-sum (DAS), which, for a given pixel, calculates the total transmit–pixel–receive path length for every transmit–receive pair, samples the signal at the corresponding time, and sums all contributions together to form the pixel value [28], [30].

The DAS reconstruction on the multistatic dataset **S**(t) is defined for a single receive channel as

(5) $$𝔻\{S(t),r\}=\sum_{i=1}^{N}a(r)s_{i}\left(\tau_{i}(r)\right)\;\tau_{i}(r)=\frac{\left|d_{i}(r)\right|+\left|d_{Rx}(r)\right|}{c}$$

where *r* is the location of a point in the imaged region, *N* is the total number of transmitting elements indexed with *i*, and *a*(*r*) is the dynamic receive apodization applied for point *r*. The delay function *τ_i_*(*r*) takes the distance from transmitting **element** *i* to point *r* (*d_i_*(*r*)) back to the receive element (*d_Rx_*(*r*)) and converts it to time, where *c* is the speed of sound in the medium. The form of *τ_i_* (*r*) is dependent on the transmission focusing scheme.

To beamform the FORCES dataset, we simply apply the DAS operator (5) to the decoded dataset (4)

(6) $$𝔽\{G(t),r\}=𝔻\left\{H_{N}^{-1}G(t),r\right\}\;𝔽\{G(t),r\}=\sum_{i=1}^{N}\left\{\sum_{e=1}^{N}{\hat{h}}_{ie}^{\left(N\right)}g_{e}\right\}\left(\tau_{i}(r)\right)$$

where ${\hat{h}}_{ie}^{\left(N\right)}$ is entry (*i*, *e*) in $H_{N}^{-1}$, and *g_e_* is the encoded signal from **event** *e*. We refer to 𝔽{**G**(*t*), *r*} as the “FORCES reconstruction operator.”

#### 3) Benefits and Challenges of FORCES:

The primary benefit of FORCES compared to other 2-D STA sequences is the increased SNR from the Hadamard aperture encoding. By using all *N* elements during each transmit event, the SNR improves by a factor of $\sqrt{N}$. TOBE arrays also enable elevational transmit focusing/steering. Since Hadamard apodization is implemented by applying DC biases to the lateral contacts, the transmitted signals can be routed to the elevational contacts with a fixed-delay focus. This delay profile [illustrated in the first event of Fig. 2(b)] ensures that the maximum amount of energy is transmitted into the plane of imaging.

The row–column symmetry of TOBE arrays allows the imaging plane to be rotated by 90° without moving the array. This enables “cross-plane” FORCES sequences in which the transmit and receive directions swap between frames or transmission events, yielding two orthogonal high-resolution images. In addition, the elevational focus can be “walked” across the array to form a 3-D volume from a stack of FORCES images. This “walking” is not achievable on standard RCAs, as their lack of bias control prevents the simultaneous implementation of lateral Hadamard transmit apodization with elevational transmit delay. Cross-plane and walking FORCES have previously been compared with volumetric imaging schemes such as linear array mechanical scanning [15], RCA-based plane and diverging wave sequences [5], and explososcan on a fully wired array [4]. In all cases, FORCES is shown to produce images with improved resolution, higher SNR, and lower sidelobe levels.

The large number of transmit events per FORCES image, combined with events mixing during aperture decoding, can result in significant motion artifacts. A sparse transmit version of FORCES, termed uFORCES, has been proposed [8]. However, the resulting images have a drastically reduced SNR and penetration compared with FORCES. Another method to compensate for motion is desired that maintains FORCES’ high-quality structural images while also resolving motion over a large region. This is the motivation for READI and EMC^2^.

## III. Methods

This section introduces the theory of READI decoding and beamforming, which yield multiple low-resolution images. It then describes the process of estimating and compensating for the underlying motion, named EMC^2^. Finally, it details the implementation of the two algorithms on NVIDIA GPUs.

### A. READI Decoding and Beamforming

READI reduces the effect of motion blurring in Hadamard aperture-encoded sequences by beamforming subsets of the complete ensemble taken over smaller periods. This will be derived and tested in the context of FORCES but can be applied to any Hadamard aperture-encoded sequence.

Due to its recursive definition [see (2)], a rank *N* Hadamard matrix can be expressed as a Kronecker product of rank *S* and *Q* Hadamard matrices, provided *S* and *Q* are both powers of two [29]

(7) $$H_{N}=H_{S}⊗H_{Q}\;H_{N}^{-1}=H_{S}^{-1}⊗H_{Q}^{-1}\;N=QS$$

where ***H***_*S*_ and ***H***_*Q*_ are smaller Hadamard matrices that form ***H***_*N*_ while $H_{N}^{-1}$, $H_{S}^{-1}$, and $H_{Q}^{-1}$ are their inverses.

According to the definition of the Kronecker product, any individual element of ***H***_*N*_ (denoted as $h_{e,i}^{\left(N\right)}$) can be expressed as the scalar product of an element from each submatrix

(8) $$h_{e,i}^{\left(N\right)}=h_{\left(q+Q(s-1)),(v+Q(l-1)\right)}^{\left(N\right)}=h_{s,l}^{\left(S\right)}h_{q,v}^{\left(Q\right)}$$

where $h_{s,l}^{\left(S\right)}$ and $h_{q,v}^{\left(Q\right)}$ are individual elements of ***H***_*S*_ and ***H***_*Q*_, respectively.

To apply this definition to FORCES, we divide the *N* transmit events sequentially into *S* groups of *Q* transmit events. The transmit **event** *e* is thus reindexed by its group number *s* and its index within the group *q*. Similarly, each transmit **element** *i* reindexed by its group number *l* and its index within the group *v*

(9) e=q+Q(s−1)i=v+Q(l−1).

With the new event indexing, we reshape the 1 × *N* FORCES dataset **G**(t) into a *Q* × *S* matrix **G’**(t), where each column contains the data from one group of *Q* transmit events

(10) $$G(t)\in {\mathbb{R}}^{N\times 1}\to G^{\prime }(t)\in {\mathbb{R}}^{Q\times S}\;{g^{\prime }}_{s}(t)≔{\text{col}}_{s}G^{\prime }(t),\;(s=1,\ldots ,S)$$

where ${g^{\prime }}_{s}(t)$ is a column vector containing the *Q* signals from the *s*’th group of transmit events.

Applying (7)–(10) to the FORCES reconstruction operator (6) yields

(11)

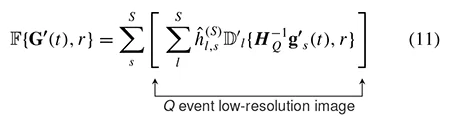


where ${\hat{h}}_{l,s}^{\left(S\right)}$ is an element of $H_{S}^{-1}$ and ${{𝔻}^{\prime }}_{l}$ is a modification of the DAS operator to account for the grouping of transmit elements. The derivation of this equation is provided in Appendix I (see Supplementary Material).

This new form of FORCES reconstruction produces a low-resolution image from each group of transmit events, then sums them to form the high-resolution FORCES image. We have named the term in square brackets that generates low-resolution images the **READI reconstruction operator**

(12a) $$𝔽\left\{G^{\prime }(t),r\right\}=\sum_{s}^{S}\mathbb{R}\left\{{g^{\prime }}_{s}(t),r\right\}$$

(12b)

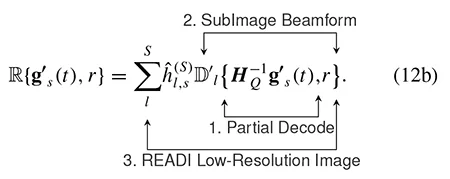



We interpret the READI reconstruction operator as consisting of three stages.

1. Partially decode of the *s*’th group of *Q* transmit events with $H_{Q}^{-1}$.
2. Beamform the partially decoded event group into *S* subimages, one for each element group *l*.
3. Sum these subimages into a READI low-resolution image, weighted with the *s*’th row of $H_{S}^{-1}$.

Fig. 2(c) highlights the READI beamforming process and contrasts it with STA and FORCES [Fig. 2(a) and (b)]. The same FORCES transmit sequence is broken into *S* = 4 groups of *Q* = 2 transmit events. Each group is partially decoded with $H_{2}^{-1}$ into a set of sparse subapertures (stage 1), then beamformed into a READI low-resolution image using (12b) (stages 2 and 3). The four READI images are then summed to obtain the final image, which is identical to the output of the standard FORCES method. Note that each partially decoded group has a different polarity pattern across its active transmit elements. These patterns correspond to the rows of ***H***_4_, and are the source of each group’s unique READI artifacts, discussed later.

Critically, each READI low-resolution image is only formed by one group **g’**_s_(t) of *Q* transmit events, rather than the complete set of *N* events. Therefore, any motion artifacts present in the FORCES image will be reduced by a factor of *N*/*Q*. This allows a single transmit sequence to produce a high-resolution image and a set of low-resolution images with good motion fidelity. As the READI operator is a linear reorganization of the FORCES operator, the compounded READI images should be mathematically identical to the FORCES image, within floating-point precision. Appendix II (see Supplementary Material) analyzes (12) to demonstrate how this perfect reconstruction is achieved.

### B. Estimated Motion-Compensated Compounding

Any feature that changes position between READI images will appear blurred in the FORCES image after compounding. If, instead, the *S* READI images are warped to align with one another before compounding, they can be summed to form a high-quality FORCES image without motion artifacts. We refer to this new process as EMC^2^.

**Fig. 3.**
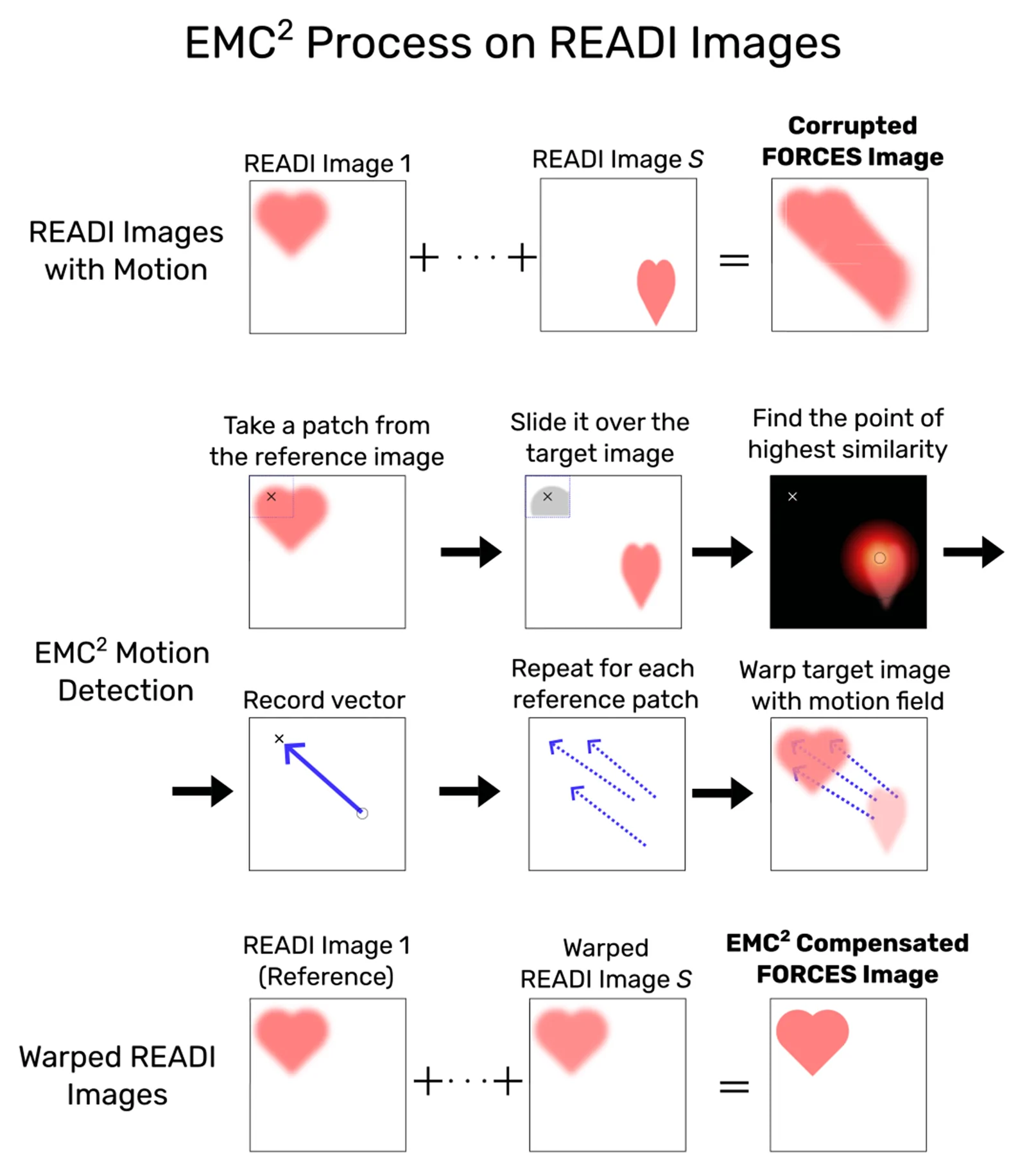
Example EMC^2^ motion compensation process. The first READI image is taken as the reference and not warped.

EMC^2^ consists of two steps: motion detection and warping. The READI images are compared to one another using a motion detection algorithm, yielding a motion vector for each point in each image. The images are then warped using these motion vectors to align them spatially before being compounded into the FORCES image. This yields an image that is both high-resolution and robust to motion blur. The basic process is illustrated in Fig. 3. EMC^2^ can use any motion-detection algorithm that operates on a sequence of beamformed images. For this work, we select normalized cross-correlation (NCC)-based block matching [31] due to its robustness to local brightness and contrast changes [32], but many other options are available and will be explored in future work.

Table I summarizes the benefits and drawbacks of the methods discussed in Sections II and III.

### C. Implementation

All beamforming and motion compensation is performed on NVIDIA GPUs using the CUDA [33] platform. The application utilizes custom CUDA kernels for beamforming and motion estimation. In addition, the cuBLAS [34], cuFFT [35], and NVIDIA performance primitives (NPP) [36] libraries provide well-optimized kernels for matrix multiplication, Fourier transforms, and image comparison, respectively. Array details are provided later with the results.

#### 1) Acquisition Rate:

All data was collected on a Verasonics Vantage 256 system with custom DC-biasing electronics [5], [37] and transferred to the CUDA application for processing. The pulse repetition frequency (PRF) of FORCES acquisitions is a combination of round-trip time to the maximum depth (2*t_z_max__*) and the time it takes for the biasing electronics to switch states (*t_bias_*). The frame rate is determined by that and the number of transmissions per frame (*N*)

(13) $$f_{\mathrm{forces}}=\frac{f_{prf}}{N}=\frac{1}{N\left(2t_{z_{\max}}+t_{\mathrm{bias}}\right)}.$$

Applying READI multiplies this frame rate by the number of groups *S*. Currently implemented electronics have a maximum switching time of 250 *μs* [37], limiting the maximum PRF to ~3 kHz and FORCES frame rate to ~24 frames/s for a 128 × 128 element array at an imaging depth of ~60 mm. Breaking up the sequence into groups of 8 with READI would increase this limit to ~375 frames/s. A new design of these electronics under development aims to reduce this delay by an order of magnitude. Experiments in this work use PRFs of 0.5–3.5 KHz at various imaging depths.

#### 2) READI Beamformer:

The READI beamforming process performs the following steps.

1. Select *S* and *Q* such that *N* = *SQ*, break the data into *S* sequential groups of *Q* transmissions.
2. Partially decode each group (${g^{\prime }}_{s}\left(t\right)$) with $H_{Q}^{-1}$ using cublasSgemm matrix multiplication.
3. Hilbert transform the partially decoded data to obtain the RF analytic signal [38].
  - Take the real to complex Fourier transform of the signal (using cuFFT).
  - Zero all negative frequency bins and double all positive frequency bins (using a custom kernel).
  - Take the complex to complex inverse Fourier transform of the modified spectrum (using cuFFT).
4. Apply the READI reconstruction operator [see (12b)] to each partially decoded group (custom kernels).
  - Beamform the group ${g^{\prime }}_{s}\left(t\right)$ into *S* subimages, together containing all in-focus terms plus extra cross terms.
  - Create a sum of these subimages weighted by the *s* ’th row of $H_{S}^{-1}$ to form the low-resolution READI image.
5. Sum the low-resolution READI images from all groups to form the final FORCES image.

See Appendix II (see Supplementary Material) for an explanation of step “D.” Both the low-resolution READI images and the full FORCES image are stored in their complex RF form for further processing.

Optionally, each pixel is weighted by its coherence factor during beamforming to improve SNR. This is a method for estimating the coherence between each sample *S_k_* forming the pixel [39]. It is defined as

$$CF=\frac{{\left|\sum S_{k}\right|}^{2}}{\sum {\left|S_{k}\right|}^{2}}.$$

As the coherence factor is a nonlinear operation, applying it to the READI low-resolution images during beamforming yields a slightly different result than applying it directly during FORCES beamforming. Accordingly, it is disabled when appropriate for comparison.

#### 3) Motion Compensation:

Our EMC^2^ implementation of block matching utilizes a CUDA-based version of NCC [31] from the NPP image processing library. One of the *S* images is selected as the reference, and all other images (targets) are compared with it using NCC. Motion is estimated at points with 4–16 pixel spacing to reduce computational cost; the output motion field is then interpolated up to the image’s resolution before warping.

As shown in Fig. 3, at each location in the motion grid, a patch is extracted from the reference image centered on the point. This reference patch is slid over a larger region of the target image, and the correlation is computed at each position. This outputs a similarity map of values in the range [−1, 1], with 1 indicating a perfect match. The peak value in this map corresponds to the location in the target image that best matches the reference patch. The motion value at this location is the vector from the peak to the center of the reference patch.

The correlation map for each reference patch is calculated with the NPP library’s nppiCrossCorrValid_NormLevel method. The peak of each similarity map is identified using a custom CUDA kernel; the subpixel peak location is obtained by fitting a 2-D paraboloid to a 3 × 3 region centered on the peak value [40].

While NCC is resilient to changes in local brightness and contrast [32], it is still susceptible to noise corrupting the comparisons. To reject spurious motion vectors, three criteria are applied to the correlation surface around the detected peak.

1. The peak value must exceed a predefined absolute threshold (reject guessing in noisy regions with weak correlation).
2. The peak must also be a set percentage above the nonmotion value (reject motion if the match is only slightly stronger than the nonmotion).
3. The curvature of the fit paraboloid must exceed a minimum (reject flat surfaces where exact motion is hard to guess).

After this process, each motion field is interpolated up to the image resolution and used to warp its source image. Summing these warped images produces a full FORCES image with drastically reduced motion artifacts.

Table II lists the parameters used for motion detection and their ranges. These parameters were manually tuned for each experiment to maximize contrast and image sharpness in motion-corrupted regions, guided by comparisons with the static cases and subjective visual quality. These metrics are described in more detail in Section IV.

## IV. Results

All FORCES data was collected using a 128 × 128 element lambda pitch TOBE array produced by CliniSonix Inc., Edmonton, AB, Canada, on a Verasonics Vantage 256 system with CliniSonix DC biasing electronics [5]. Fig. 1(d) provides a block diagram of this setup. Table III lists the imaging parameters. These are valid for all experiments unless otherwise noted.

Three primary metrics are used to evaluate the results: generalized contrast-to-noise ratio (gCNR), speckle similarity, and image sharpness. gCNR [41] measures the overlap between the probability density function of pixels within a contrast region and that of pixels in a background speckle region. A gCNR of 1 indicates no overlap between these distributions and perfect contrast, while a gCNR of 0 indicates complete overlap and no contrast. This method is also used to compare the speckles of the corrupted and EMC^2^-compensated images; here, the scale is flipped so that 1 indicates complete overlap of the probability densities (and thus recovery of the original speckle distribution). We refer to this comparison as “speckle similarity” in the results. Finally, image sharpness is measured from the spatial Fourier domain [42]. It is calculated as the ratio of significant frequency components to the total number of frequency components. Sharp images will contain contributions from a wide range of frequencies, whereas blurry images will contain fewer significant frequencies. Speckle similarity is calculated on the linear beamformed images; all other methods are calculated after log-compression.

### A. Field II Simulations

Multiple simulations were performed with Field II [9], [10] to validate the READI beamforming process and to explore the effects of motion and resolution; their results are collected in Appendixes III-A and III-B (see Supplementary Material). First, the READI beamformer was validated with a static 128 transmit FORCES point-spread function simulation. At all tested group sizes (*Q* = 32, 16, 8), the compounded READI image was identical to the FORCES image within floating-point precision, confirming the mathematical equivalence of the two operators discussed in Section III-A. Resolution was measured by simulating the point spread function at various depths. The READI images maintain the lateral resolution of FORCES even with few transmits per group, but do show some decrease in axial resolution and sidelobe height as group size decreases. These results are included in Appendix III-A in the supplementary materials.

Appendix III-B (see Supplementary Material) provides simulations of the effects of motion on READI images, demonstrating that small READI group sizes can significantly improve resolution and enable motion estimation even when the FORCES image is completely corrupted. Simulation parameters are also provided with their respective results. All simulation code is available upon request. Coherence factor weighting was disabled for these noiseless simulations to preserve linearity, but is enabled for all subsequent results.

### B. Static Comparison

**Fig. 4.**
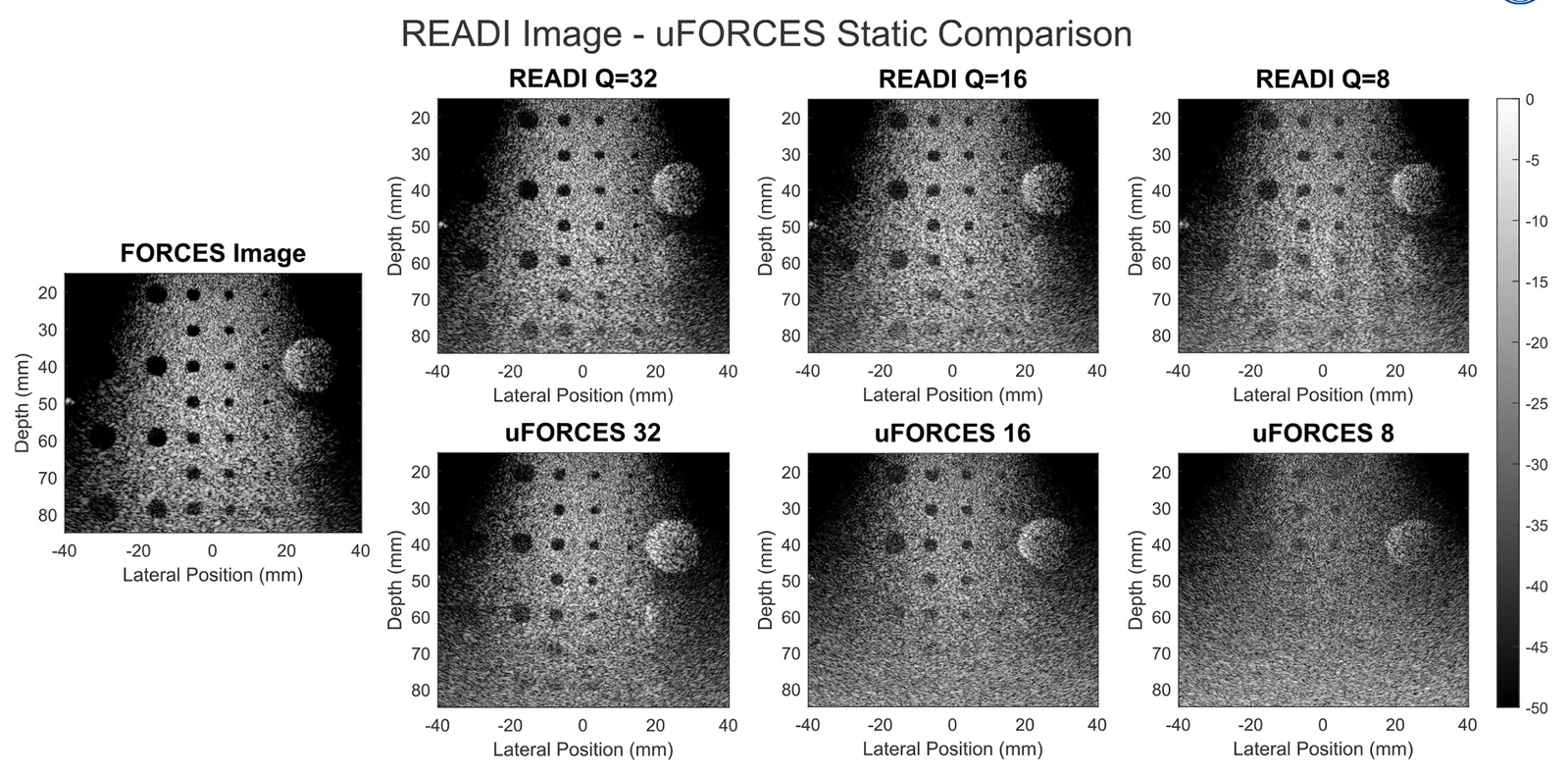
Comparison between READI low-resolution images and uFORCES images with the same transmit count. (Left to right) 128 transmit FORCES image, 32 transmit comparison, 16 transmit comparison, and 8 transmit comparison.

For the first real-world experiment, an anechoic cyst phantom (*ATS 539*, CIRS Inc., Norfolk, VA, USA) was imaged with FORCES and beamformed again with READI using group sizes of 32, 16, and 8. The quality of the READI low-resolution images was compared with uFORCES images with the same transmit count. uFORCES is a sparse transmission version of FORCES that uses *Q* < *N* Hadamard encoded transmissions [8]. The decoded uFORCES dataset contains *Q* − 1 signals, each representing a single transmitting element. The leftover signal contains the contributions of all other *N* – *Q* + 1 elements and is discarded. Fig. 4 compares the first READI image at each group size to a uFORCES image formed with the same transmit count. Both image sets degrade as the transmit count decreases, but at each transmit count, the READI image exhibits a visibly higher SNR than the uFORCES image.

**Fig. 5.**
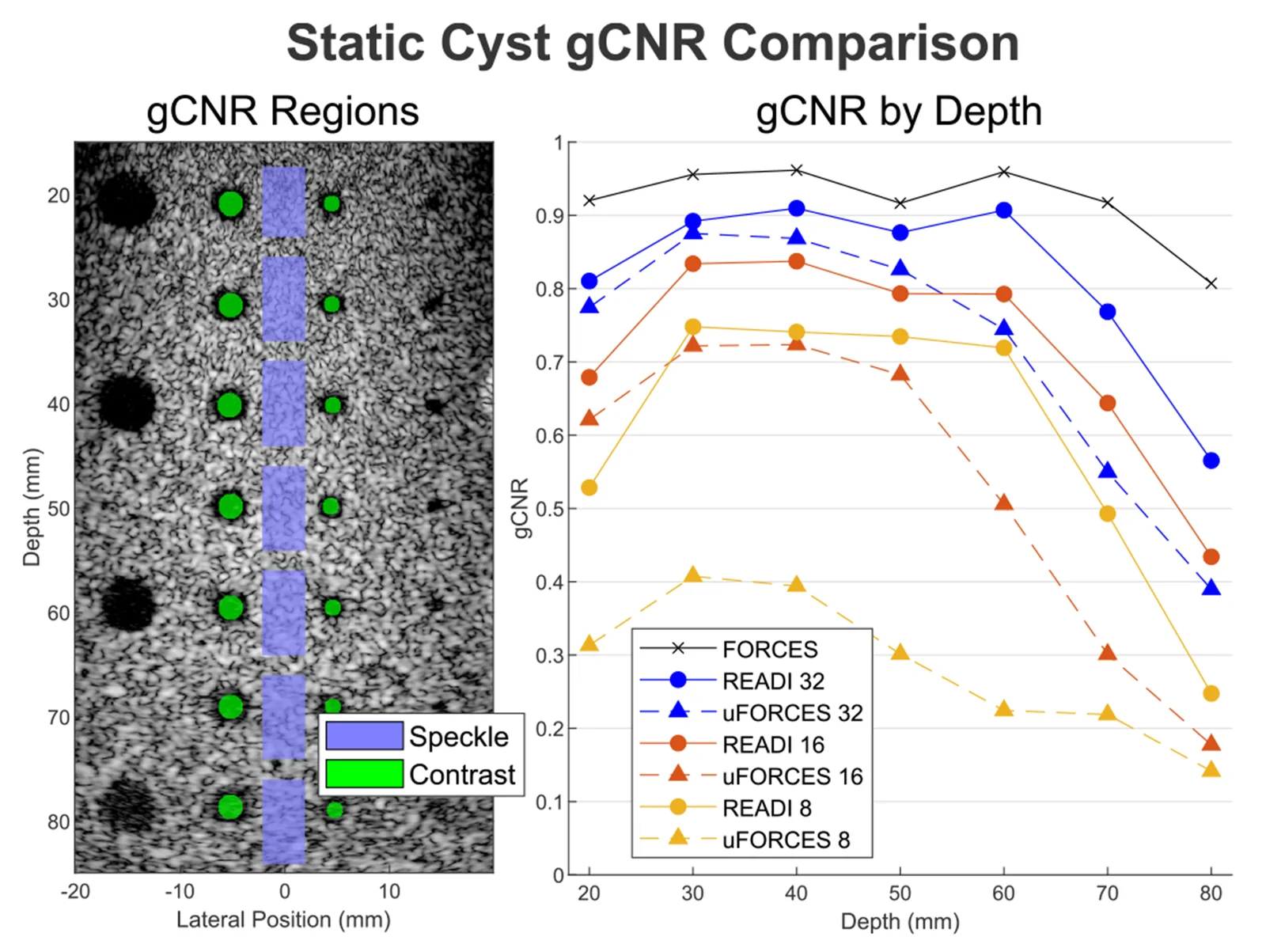
gCNR measurements for the static cyst phantom comparison, regions of interest for each depth are marked on the left.

To quantify this quality difference, the gCNR was computed for the middle two columns of cysts at each depth. The results for the seven images are plotted in Fig. 5 with the regions of interest marked. The READI images consistently outperform uFORCES at all depths and transmit counts, with the gap increasing as transmit count decreases.

### C. Probe Motion

**Fig. 6.**
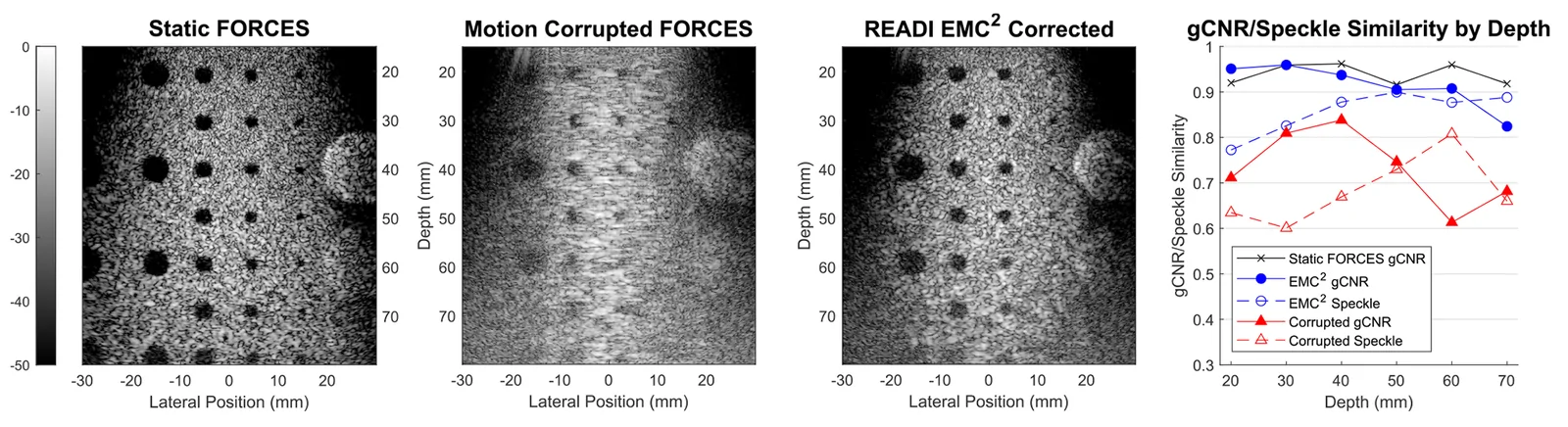
FORCES image before (left) and after (center) EMC^2^ motion compensation. (Right) gCNR measurements (solid lines) and speckle similarity (dashed lines) at each depth. Speckle similarity is calculated by comparison with the static case.

**Fig. 7.**
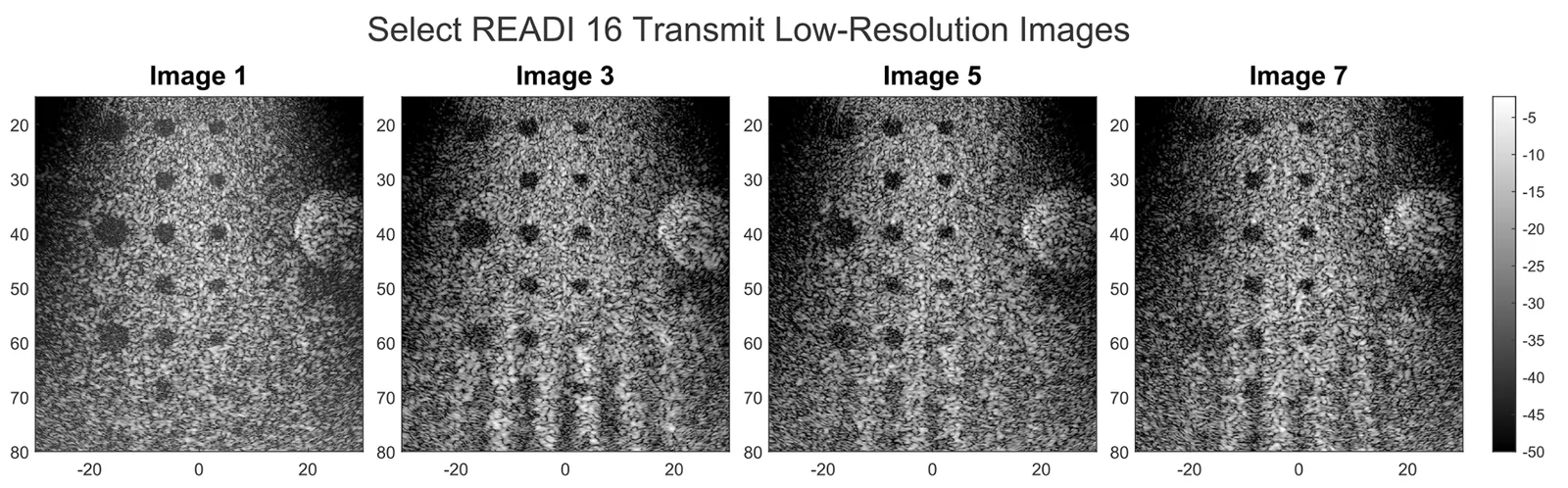
Select READI low-resolution images from the probe motion experiment. All images are formed from groups of 16 transmissions.

Next, the cyst phantom was imaged while the operator manually moved the probe laterally at an approximate speed of 18.5 mm/s. This resulted in a significantly corrupted FORCES image where the cysts are barely visible. The data was then split into eight groups of 16 transmissions (*Q* = 16, *S* = 8) and beamformed using READI; EMC^2^ was then applied to the eight low-resolution images. The EMC^2^ compensated image shows a significant reduction in motion artifacts, with the cysts and background speckle clearly visible (Fig. 6). Fig. 7 shows select READI low-resolution images from this experiment. Each group has a unique brightness pattern introduced by READI decoding that appears as vertical striping. These READI artifacts worsen with depth and are more severe for small group sizes.

The gCNR was measured for each image at the same locations marked in Fig. 5, with results presented in Fig. 6. At each depth, the speckle of the corrupted and EMC^2^ images were also compared with the static case; their speckle similarity scores are plotted as dashed lines. The sharpnesses of the three images were also measured between −20 and 20 mm, with results in Table IV. The EMC^2^ compensated image successfully recovers the same gCNR as the static image for all cysts clearly visible in the low-resolution images. Beyond a depth of 70 mm, the cysts have been lost in the noise and READI artifacts and are unrecoverable. The EMC^2^ recovered speckle also shows improved similarity with the static case at each depth. Averaging the estimated motion between each of the eight READI images yields an estimated average lateral speed of 17.6 ± 1.3 mm/s, which agrees with the expected value of 18.5 mm/s within error.

This demonstrates that EMC^2^ can recover a B-mode of similar quality to a static FORCES image in the presence of bulk motion. Comparing the EMC^2^ compensated cyst image with the static FORCES image, the leftmost column of large cysts is outside the compensated image’s field-of-view. While EMC^2^ successfully resolves structures within the field-of-view, it does not return it to the width of the static case. Reducing the number of transmissions per group to *Q* = 8 and adjusting the receive apodization did not recover the lost information. This indicates that READI can easily recover information that remains within the field-of-view across the entire ensemble, but significant bulk motion can lead to information loss at the image edges.

### D. Beating Heart Phantom

**Fig. 8.**
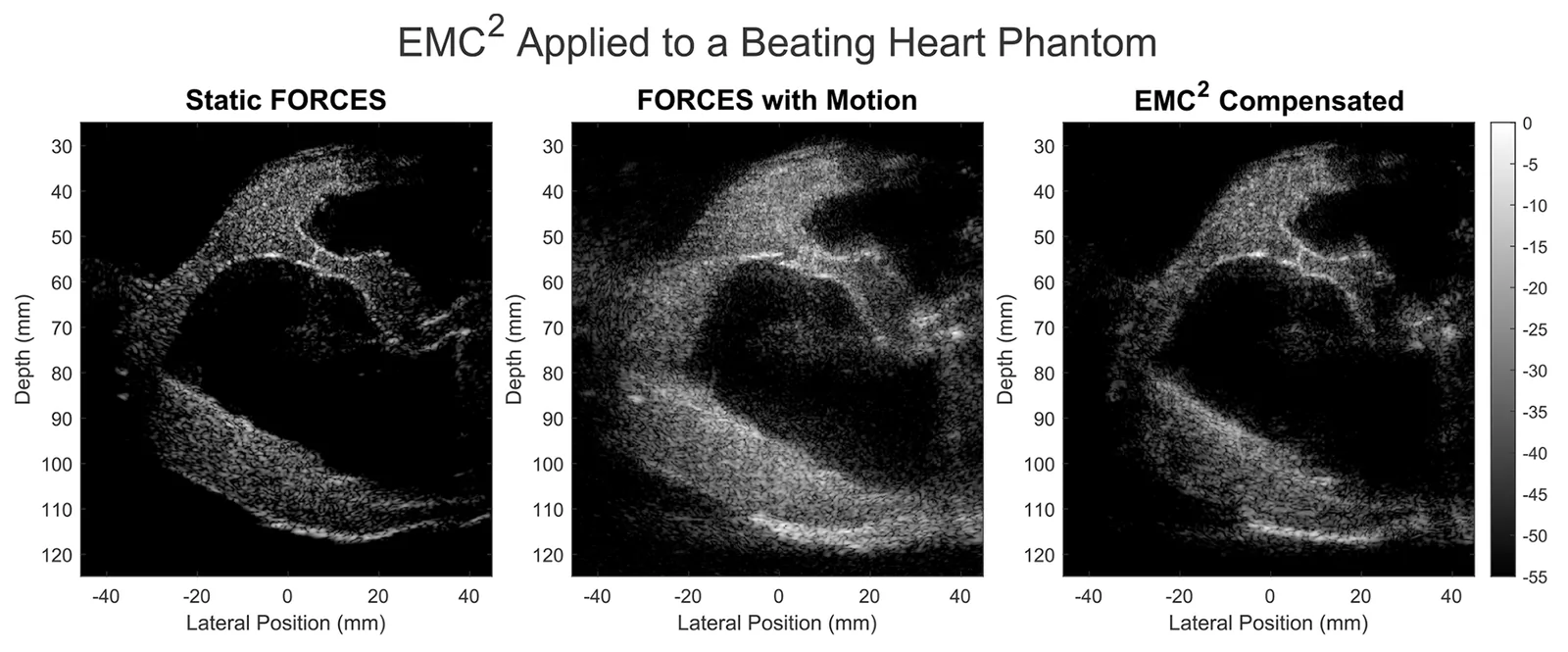
EMC^2^ compensated image of a beating heart phantom (right) compared with the motion corrupted image (center) and a FORCES image of the static heart (left).

For a more realistic test case, a heart phantom (*DHP01*, Shelley Medical Imaging Technologies, Toronto, ON, Canada) in a water bath was imaged as a servo simulated a 120 beats/min heartbeat. This experiment was performed at 5 MHz with a PRF of 0.5 kHz. The servo arm is attached to the bottom of the heart (at x = −40 mm in the images) and is actuated to simulate the beating motion. While this phantom lacks true 3-D heart motion, it serves as a useful test case for READI’s ability to resolve general tissue motion. The servo takes 0.3 s to complete a compression stroke and compresses the heart linearly by 14.3 mm. Thus, we can estimate the average lateral speed of the heart near the connection point (on the left side of the image) to be approximately (14.3/0.3) = 47.7 mm/s. During continuous motion, the phantom has a BPM of 120, leaving 0.25 s for each stroke (compression and retraction). Again READI was applied using eight groups of 16 (*Q* = 16, *S* = 8) and EMC^2^ attempted to recover the original image quality. The resulting images are compared in Fig. 8.

**Fig. 9.**
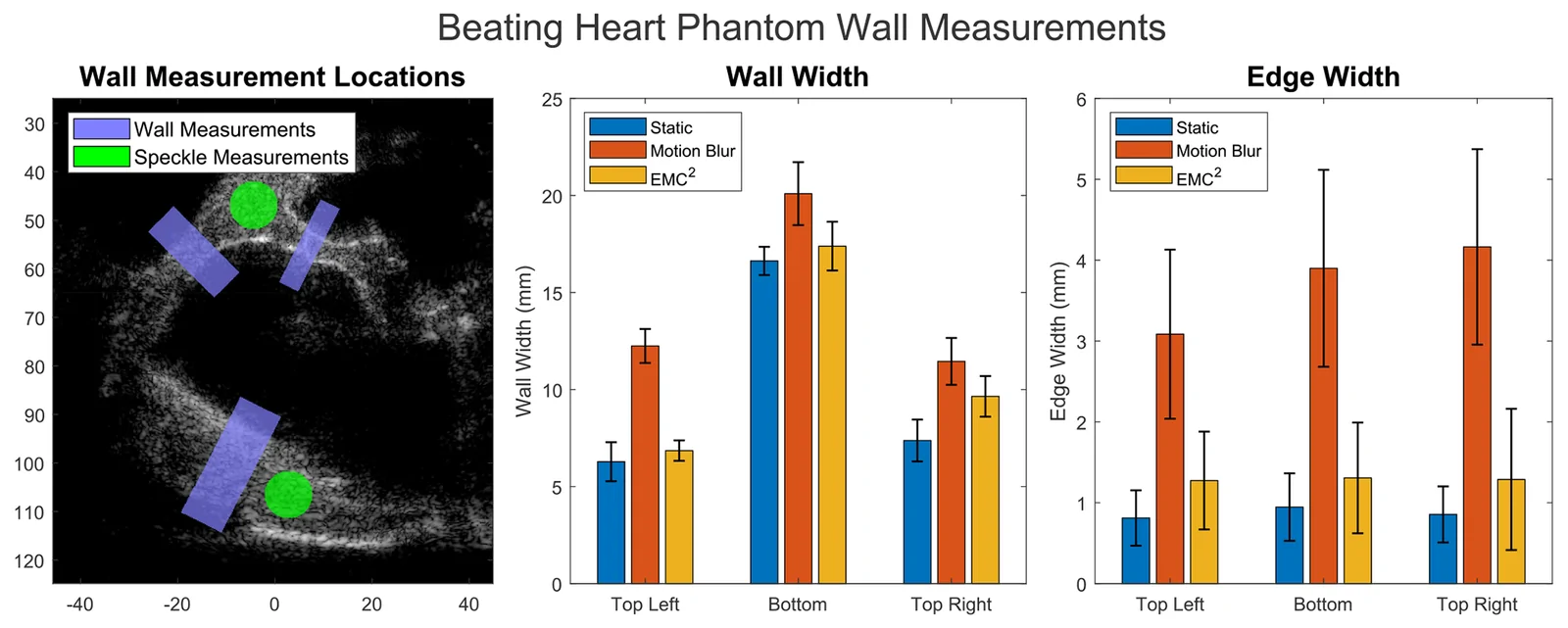
Wall width and edge thickness measurements for the marked regions on the heart phantom images in Fig. 8. Error bars indicate the standard deviation of these measurements across the marked regions.

Due to the high contrast between the heart phantom and the surrounding water, gCNR is not an ideal metric for evaluating region separation. Accordingly, gCNR was replaced with edge-thickness measurements in this experiment. The wall and edge thickness of the heart was measured at three locations. Regions with values above −40 dB are considered part of the wall, whereas regions with values below −50 dB are considered background. Edge thickness is measured as the distance from the first peak over −40 dB to the first trough under −50 dB, while wall thickness is measured as the distance between the first and last peaks over −40 dB. These measurements are plotted in Fig. 9. The motion-corrupted image exhibits significantly wider edges and walls than the static image, whereas the EMC^2^ compensated image recovers sharper edges and accurate wall thicknesses. Speckle similarity was measured at the locations marked in green on Fig. 9. It is collected in Table V along with the sharpness measurements and average thickness measurement errors. The recovered edge widths on the order of ~1 mm are comparable to the lateral resolution of FORCES at those depths (presented in Appendix III-A, see Supplementary Material).

**Fig. 10.**
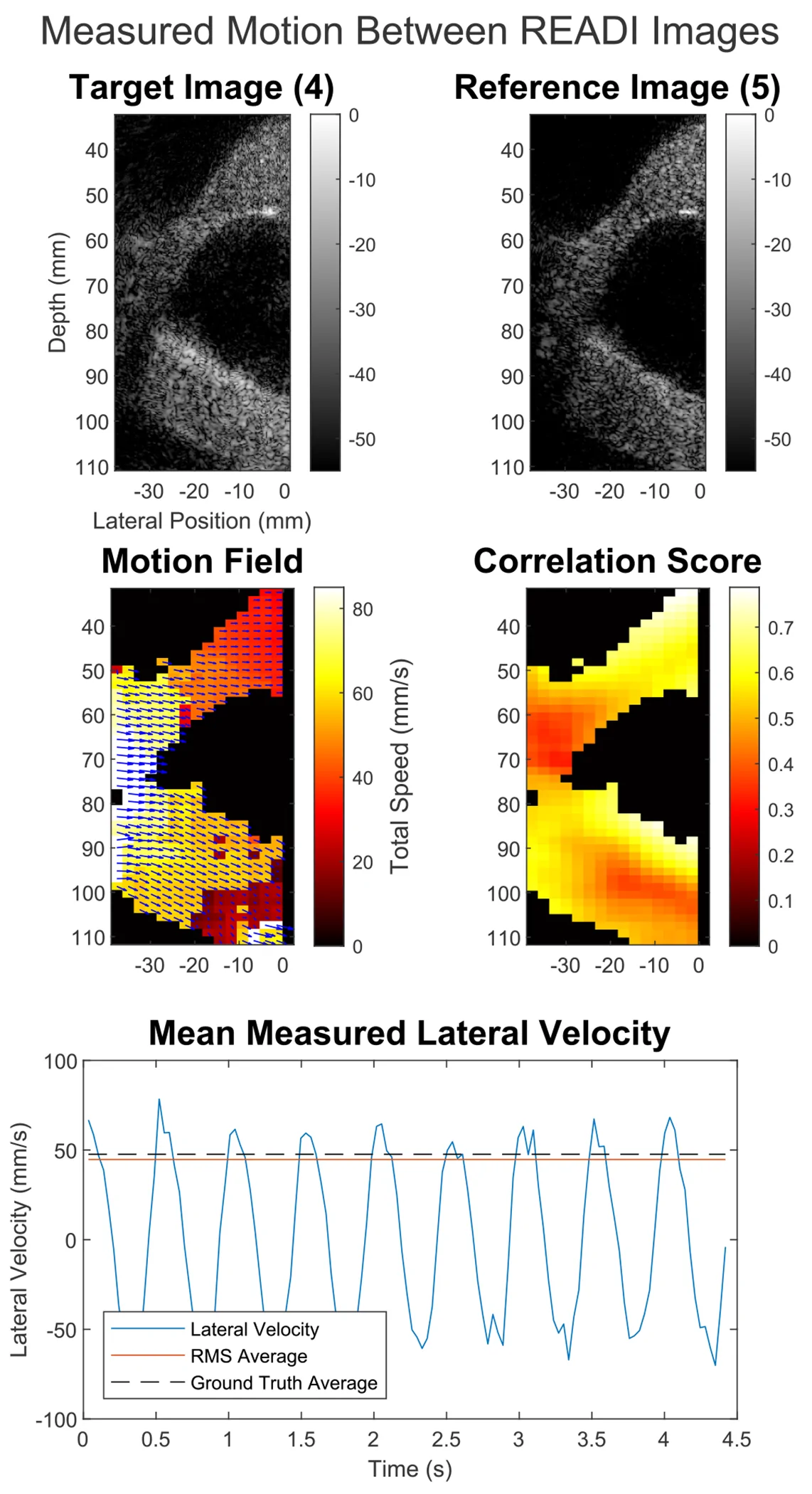
(Top) Measured motion between the fourth and fifth READI images from the heart phantom experiment, taken from the left wall of the heart. The color of the third image indicates the total speed, while the fourth indicates the correlation score of the estimated vector. (Bottom) Average lateral velocity of the phantom on the left wall, across 16 FORCES frames (128 READI images).

To assess the effectiveness of the motion tracking algorithm, each READI image is compared with its neighbor rather than a single reference. Fig. 10 shows the motion field between images 4 and 5 in the region near the servo connection, along with the correlation score for each vector. The color of the motion field background indicates the total speed in mm/s. Under this, the measured average lateral velocity at the left edge is plotted from a set of 16 FORCES images, split into 128 READI images. A clear sinusoidal pattern is visible as the heart beats, with an rms average value of 44.8 ± 1.4 mm/s and a period of 0.502 ± 0.019 s (119 ± 4 beats/min), in good agreement with the estimated ground truth averages of 47.7 mm/s and 120 beats/min. Supplementary figures are included in Appendix III-C that display all eight READI low-resolution images and the seven motion fields detected between them.

The correlation estimator successfully outputs a smooth motion field on the top and left portions of the heart. On the lower wall, however, the correlation score decreases, and the field becomes more random. The lower SNR due to attenuation and the increased READI artifacts at lower depths combine to produce noisier correlation surfaces, which strain the simple peak-detection algorithm.

An example cross-plane video of the beating heart phantom is included in the supplementary materials, comparing the original FORCES sequence to READI images without motion compensation. This is discussed further in Section V-B1

### E. Flow Phantom

**Fig. 11.**
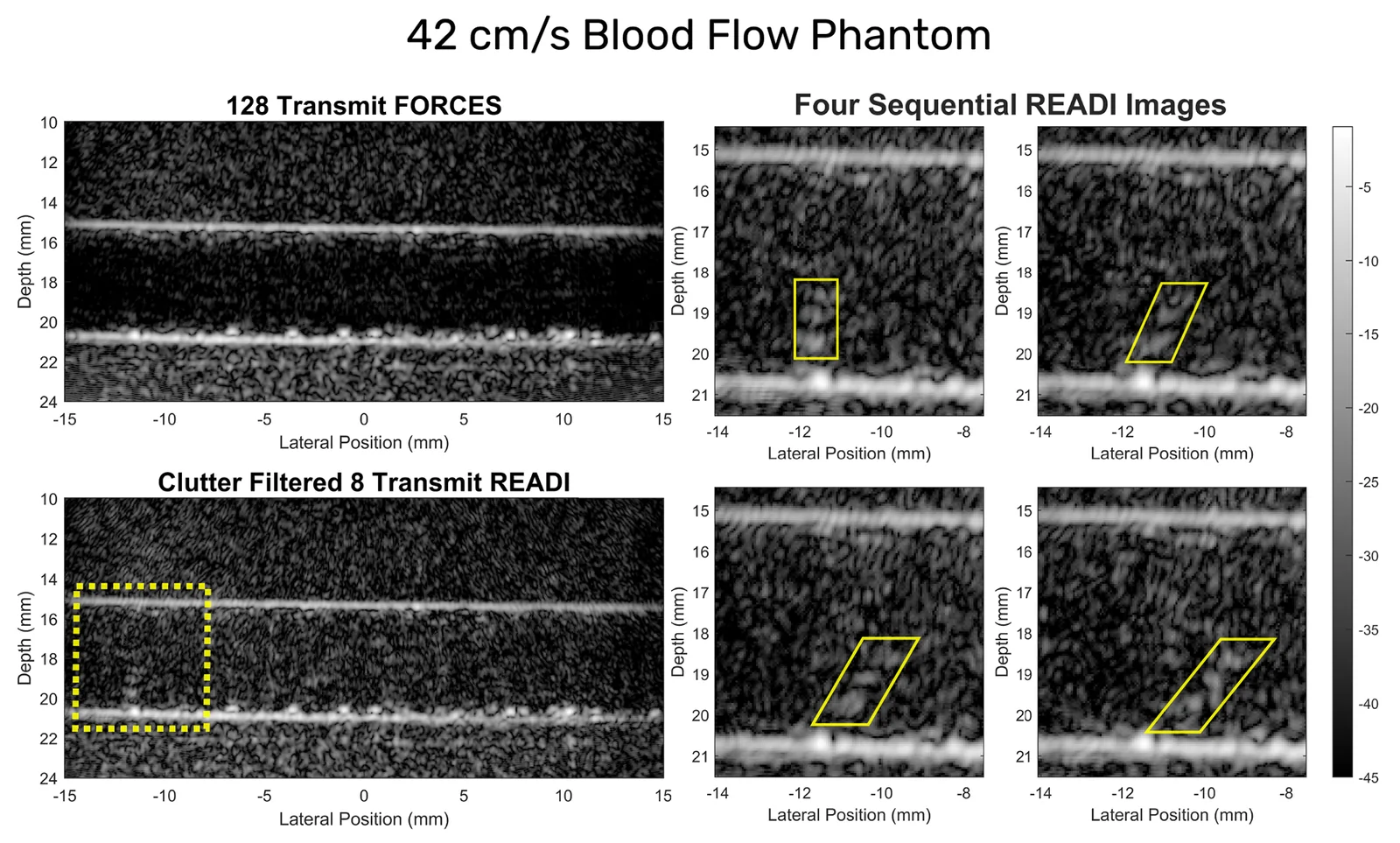
6-mm diameter blood vessel phantom imaged with READI using 16 groups of 8 transmits. (Top left) Standard FORCES Image. (Bottom left) Eight transmit READI image postclutter filtering. (Right) Four frames from the filtered READI sequence, with three speckle grains tracked.

As a final proof of concept, a 6-mm diameter blood vessel phantom (*ATS 524*, CIRS Inc., Norfolk, VA, USA) was imaged with an 8 MHz 128 × 128 element TOBE array at a PRF of 3.5 kHz and maximum depth of 25 mm. Blood-mimicking fluid was pumped through the phantom at a rate of 42 cm/s. READI was performed with 16 groups of eight transmits (*Q* = 8, *S* = 16) to improve blood speckle visibility. The results are displayed in Fig. 11. At these flow rates, scatterers can move by more than 12 mm during the FORCES sequence, preventing coherent compounding of the blood signal during beamforming. This results in a FORCES image in which the vessel appears nearly empty, with no speckle present other than the vessel walls. In contrast, the scatterer will move only 0.75 mm during an eight-transmit READI group, reducing negative interference and enabling stronger blood speckle.

The tradeoff of using smaller READI groups of eight transmits is noisier, low-resolution images with more significant READI artifacts. To remove these artifacts and isolate the flow signal, an ensemble of 16 FORCES images was acquired, and each was beamformed with READI to yield 256 low-resolution images in total. These images were clutter-filtered using basic singular value decomposition (SVD) [43]. When applying SVD to the READI sequence in Fig. 11, the largest singular value was kept to preserve the vessel walls for visualization purposes.

After clutter filtering, the blood speckle is clearly visible as it flows through the vessel, and features of parabolic flow can be observed. The right side of Fig. 11 tracks three speckle grains across four images. The motion of the grains is readily tracked between frames, and their speed differences due to parabolic flow are evident. The 42-cm/s flow meets or exceeds peak flow velocities in almost all veins, and is comfortably in the range of average larger arterial flow rates [44].

In addition to directly filtering the 256 READI frames, they were combined into 241 FORCES images using a 16-frame moving window, after which the same SVD filter was applied. While flow dynamics are observable, individual speckle grains are smeared out by the compounding process and are not easily tracked. A video comparison of the original FORCES sequence to the clutter-filtered READI and moving window FORCES sequences is included in the supplementary materials. Both clutter-filtered sequences were superimposed on top of their unfiltered backgrounds for visualization purposes. This video has been slowed to make blood flow easier to track.

## V. Discussion

### A. Next Steps

These results are extremely promising for the application of READI to many ultrasound imaging tasks and provide clear next steps for further improvement.

#### 1) Motion Detection Algorithms:

The motion field shown in Fig. 10 illustrates the limitations of the basic NCC block-matching algorithm used in this work. While it successfully tracks motion along the top and left walls of the heart, its performance degrades on the lower wall, as lower SNR and more pronounced READI artifacts reduce image correlation. Once this correlation falls low enough, a large discontinuity in the field occurs, and the vectors begin to point in a spurious direction. Meanwhile, correlation scores on the left wall drop even lower, but the field remains smooth and points in the correct direction. This demonstrates that simple correlation with a threshold may not be the optimal method for EMC^2^ motion detection.

These issues may be mitigated by implementing more robust motion detection algorithms, such as biased block matching with priors [45] or optical flow-based methods that require local motion continuity [46]. A quantitative, no-reference cost function should be developed alongside each motion detection algorithm to automatically tune parameters without a priori knowledge or user input, starting with the gCNR and sharpness metrics used in this work.

#### 2) Real-Time Implementation:

Palamar et al. [47] present an open-source beam-former for RCA and TOBE arrays. They provide an in-depth analysis of the computational requirements for Hadamard decoding, envelope detection, and DAS beamforming. They demonstrate that consumer-grade GPUs can beamform FORCES images with ~500k pixels at a rate of ~60 frames/s. This is well above the current acquisition limit of ~24 frames/s from the biasing hardware and is also limited by data transfer rates out of the Vantage system.

Applying READI multiplies the FPS by the chosen number of groups without increasing the data transfer or acquisition rate. This allows us to bypass the identified bottlenecks, enabling real-time reconstruction up to 60+ frames/s. A READI image requires the same amount of DAS operations as a FORCES image, as each of the *Q* signals is beamformed *S* times.

EMC^2^ with its current correlation-based algorithm, is too computationally expensive for real-time playback. The CUDA-based implementation of EMC^2^ is roughly 20 times slower per image than beamforming, which would limit the maximum overall frame rate to below 3 frames/s. As new motion detection algorithms are explored, one will be selected for low computational complexity and implemented in CUDA to enable real-time EMC^2^ compensation.

#### 3) READI Artifact Filtering:

READI artifacts in the low-resolution images (Fig. 7) manifest as global intensity variations that worsen with smaller group sizes, thereby lowering the correlation between sequential images and reducing the effectiveness of motion compensation. Methods to filter out these artifacts should be explored to improve READI’s performance with small group sizes.

Testing with SVD filtering during the flow phantom experiment revealed that the majority of READI artifacts are mapped to singular values between the tissue and flow bands [48]. This can potentially allow for selective filtering of these artifacts while maintaining both tissue and flow information. As a tradeoff, SVD is computationally expensive and requires a large ensemble of images to be effective. Another option is applying a method similar to flat-field correction in optical imaging, where the background brightness pattern is modeled and then divided out of the image [49].

### B. Future Work

#### 1) Volumetric Imaging With READI:

**Fig. 12.**
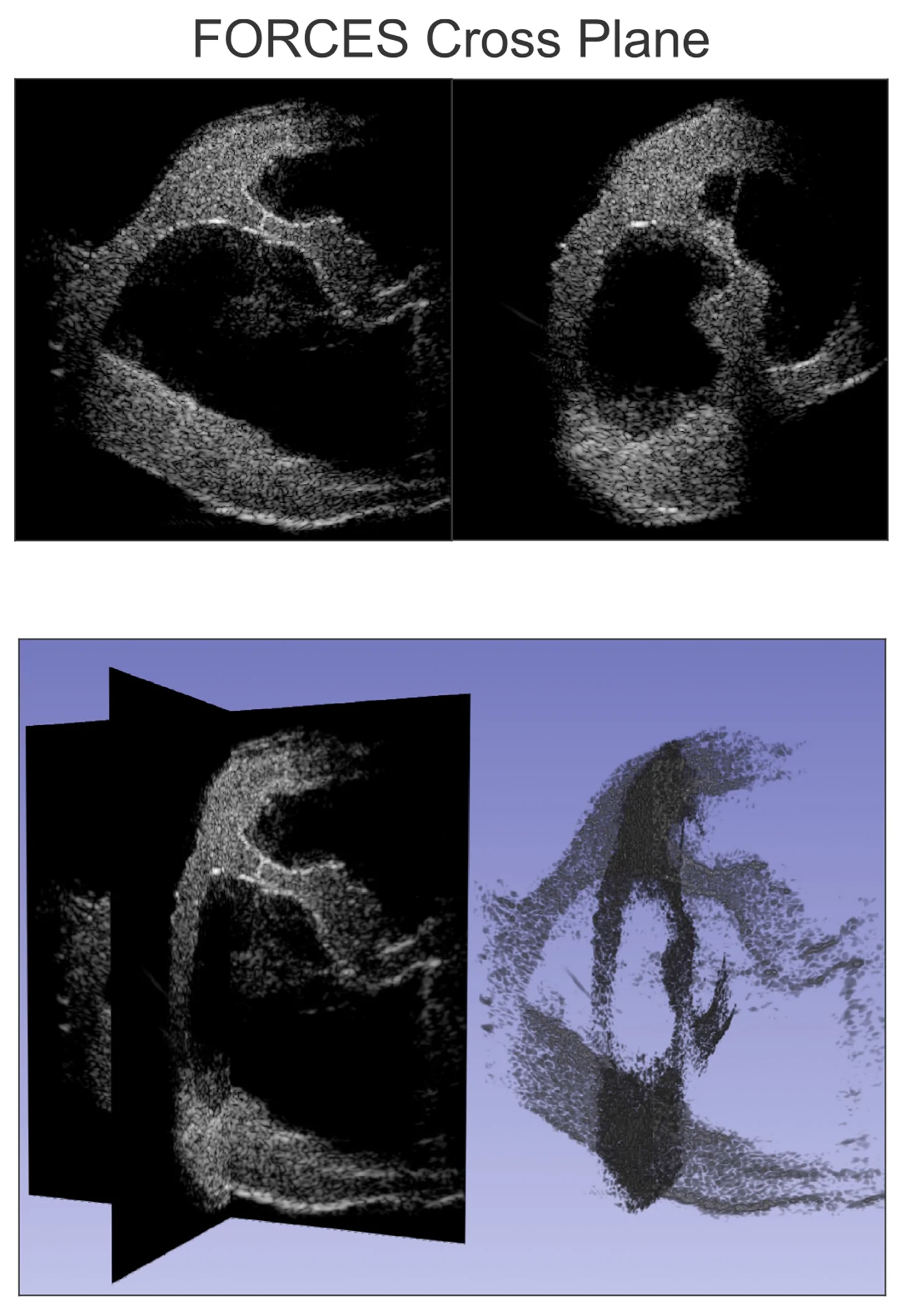
Example cross-plane volume of the heart phantom, demonstrating the 3-D capabilities of FORCES.

READI and EMC^2^ can be directly applied to the walking and cross-plane FORCES sequences discussed in the background, where READI is applied independently to each FORCES plane. Fig. 12 provides an example cross-plane image of the heart phantom, and a fly-around video of crossed planes is provided in the supplementary materials. In addition, a video of both planes beating simultaneously is also provided. In this video, the low-frame-rate FORCES sequence is compared with the high-frame-rate READI 32 sequence without motion compensation. The motion in the READI sequence is noticeably smoother than the FORCES sequence, and the loss in quality due to lower transmit counts is offset by the decreased motion blur.

The example cross-plane sequence was taken with a PRF of 1 kHz. As it interleaves two FORCES planes, each plane has an effective PRF of 0.5 kHz. With the current PRF limit of ~3 kHz, six FORCES sequences on different planes could be interleaved in the same manner. Each plane would have an effective PRF of 0.5 kHz, so its motion corruption would be comparable to the EMC^2^ heart example (Fig. 8). The width of these images (from −45 to +45 mm) extends far beyond the shadow of the aperture (from −16 to +16 mm). This is unlike most previous RCAs [5]. EMC^2^ combined with this large field-of-view enables 3-D tissue/organ characterization in the presence of motion, such as chamber volume estimation.

Beyond FORCES, the recursion and partial decoding at the core of READI apply to all Hadamard aperture-encoded sequences. HERCULES is a recently presented 3-D Hadamard encoded sequence on TOBE arrays [18] that uses the same amount of transmission events as 2-D FORCES. Rather than generating a 2-D image with global transmit and receive focusing, it produces a 3-D volume with global receive focusing in all directions. This technique suffers from the same motion sensitivity sources as FORCES, so it can benefit from READI and EMC^2^ to reduce motion artifacts.

#### 2) Flow Estimation:

**Fig. 13.**
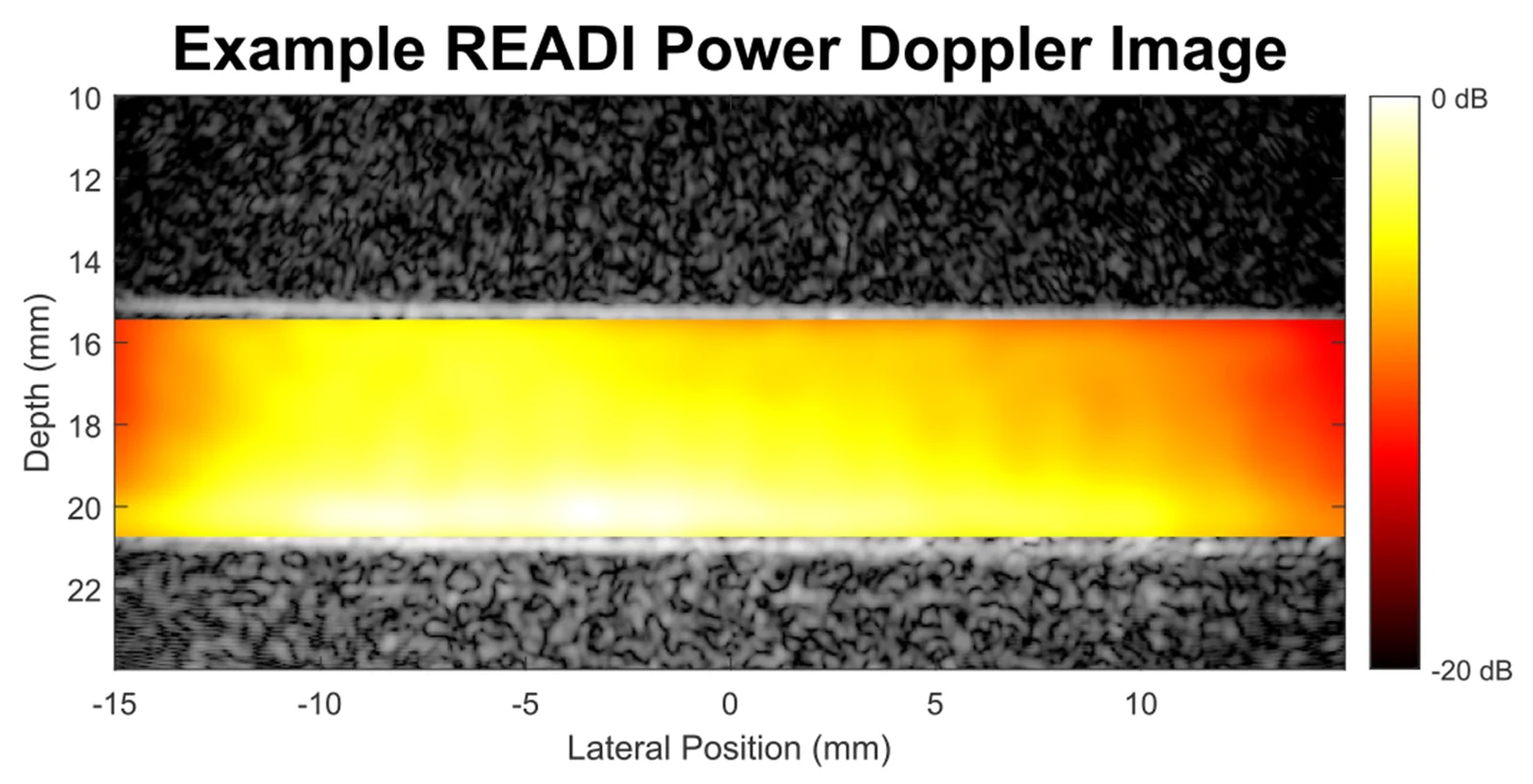
Example power Doppler image of the flow phantom, created by averaging the SVD clutter-filtered READI low-resolution images.

The success of the flow phantom experiment suggests that flow imaging techniques could be applied to READI images. While some vessels experience flow rates exceeding 42 cm/s, the simulations in Appendix II-B (see Supplementary Material) suggest that, at a PRF of 3.5 kHz, coherent lateral motion is measurable at speeds up to 188 cm/s. Currently, measurable flow velocities may be insufficient to measure pathological conditions such as carotid stenoses. New biasing electronics with higher PRF capabilities would allow for even higher flow rates to be measured. The clutter filtered READI sequence is already close to a power Doppler image, and the clear speckle flow visible in it suggests that more robust color and vector flow techniques should be applicable [50], [51]. As an example, a basic power Doppler image was created by time averaging the 256 SVD-clutter filtered READI images (Fig. 13).

Applying READI to flow imaging can produce both a high-quality B-mode image and a detailed flow map. Both datasets would be perfectly co-registered in time without the need to interleave separate B-mode and Doppler sequences. The improved edge sharpness at tissue boundaries, enabled by motion compensation, should also improve the accuracy of flow characterization by providing more precise vessel/chamber boundaries.

#### 3) Elastography:

Another imaging modality that involves motion is shear-wave elastography. This technique induces a shear wave in tissue and tracks its propagation via high-frame rate B-mode imaging [52]. Shear waves travel and attenuate rapidly in soft tissue, requiring an imaging method with a high frame rate while still producing images detailed enough to track subtle tissue deformations as the shear wave propagates. The comparison of READI images with uFORCES images of the same transmit count suggests that READI may be ideal for this application, as it maintains good structural resolution over a large field-of-view at low transmit counts. Shear-wave elastography has previously been implemented in 3-D with standard RCAs [53], so it is a good next step for READI on TOBE arrays.

## VI. Conclusion

This work presents READI with EMC^2^, a new method for beamforming Hadamard aperture-encoded sequences that reduces motion artifacts while maintaining high spatial resolution. The intermediate READI low-resolution images are shown to be a marked improvement over sparse transmit aperture uFORCES images at the same transmission count. READI with EMC^2^ successfully removed blur due to probe motion, reproducing an image of comparable quality to standard FORCES with a static probe. With a beating heart phantom, READI EMC^2^ removed speckle blur and sharpened the edges of moving tissue regions, showing its potential for tissue motion canceling and region segmentation. READI also successfully recovered blood speckle in a vessel phantom at a flow speed of 42 cm/s, demonstrating its potential for high-resolution flow imaging in large arteries and veins. Multiple avenues for further development of READI have been identified, including 3-D volumetric imaging, vector flow characterization, and shear-wave elastography. These applications will be explored in future work.

## Supplementary Material

supp3-3704971

supp1-3704971

supp2-3704971

supp4-3704971

This article has supplementary material provided by the authors and color versions of one or more figures available at https://doi.org/10.1109/TUSON.2026.3704971.

## Figures and Tables

**TABLE I:** STA Method Comparison

Method	Pros	Cons
Base STA	High Resolution, Fast, Simple Implementation	Low SNR. Bad Penetration, Sensitive to Motion
FORCES	High Resolution and SNR, Good Penetration, Fast	Sensitive to Motion
uFORCES	Ultrafast, Low Motion Sensitivity	Low Resolution and SNR, Bad Penetration
READI with EMC^2^	High Resolution and SNR, Good Penetration, Fast, Robust Motion Compensation	Complex Beamforming

**TABLE II:** NCC Motion Detection Parameters

Motion Grid Size	4-16 px
Reference Patch Size	16-64 px
Search Patch Size	Ref. size + 8-64 px
Absolute Peak Threshold	0.01 - 0.5
Relative Peak Threshold	101% - 120%
Minimum Curvature	0.005 - 0.1

**TABLE III:** Imaging Parameters

FORCES Transmit Count	128
Transmit Frequency	4.3 MHz
Element Pitch	*λ*
Transmit Waveform	2-cycle sinusoid
Pulse Repetition Frequency (PRF)	1 kHz
Dynamic Rx Apodization F#	1
Coherence Factor Weighting	Enabled
Pixel Size	0.05 × 0.05 mm

**TABLE IV:** Cyst Sharpness Comparison

Image	Sharpness	% Error
Static FORCES	0.106	N/A
Blurred FORCES	0.045	58%
EMC^2^ Compensated	0.069	35%

**TABLE V:** Beating Heart Phantom EMC^2^ Performance

Metric	Static FORCES	Blurred FORCES	EMC^2^ Compensated
Sharpness	0.099	0.046 (−53.3%)	0.094 (−5.2%)
Speckle Similarity	N/A	0.688	0.812
Average Wall Error	N/A	59.0%	11.1%
Average Edge Error	N/A	316.4%	32.5%

## Highlights

- Hadamard-encoded sequences can be recursively decoded into subgroups with fewer motion artifacts. These subgroups form low-resolution images that can be compounded to form a high-resolution image.
- Applying motion compensation before compounding yields a motion-robust high-resolution image.
- This allows for ultrafast imaging with reduced motion artifacts and improved flow detection using only B-mode images.

## Nomenclature

DAS: Delay-and-sum
EMC^2^: Estimated motion-compensated compounding
FORCES: Fast orthogonal row–column electronic scanning
gCNR: Generalized contrast-to-noise ratio
HERCULES: Hadamard encoded row–column ultrasonic expansive scanning
NCC: Normalized cross correlation
NPP: NVIDIA performance primitives
PRF: Pulse repetition frequency
RCA: Row–column array
READI: Recursive aperture decoded ultrasound imaging
SNR: Signal-to-noise ratio
STA: Synthetic transmit aperture
SVD: Singular value decomposition
TOBE: Top-orthogonal-to-bottom-electrode
uFORCES: Ultrafast FORCES
