# Supplementary material for: Hadamard-Based Recursive Aperture Decoded Ultrasound Imaging (READI) With Estimated Motion-Compensated Compounding (EMC2) Using Top-Orthogonal-to-Bottom-Electrode (TOBE) Arrays
Source: IEEE Trans Ultrason. Author manuscript; Available in PMC 2026 Sep 15. (PMC13576084; doi:10.1109/TUSON.2026.3704971)
Supplement: Supplementary file 1 [file NIHMS2205368-supplement-supp1-3704971.pdf]

APPENDIX I
READI DERIVATION

This section provides a detailed derivation of the READI reconstruction operator (eq. 12) from the base FORCES reconstruction operator (eq. 6):

Starting point:

$$\mathbb{F}\{\mathbf{G}(t), r\} = \mathbb{D}\{\boldsymbol{H}_N^{-1}\mathbf{G}(t), r\}$$

Goal:

$$\mathbb{R}\{\mathbf{g'}_s(t), r\} = \sum_l^S \hat{h}_{l,s}^{(S)} \mathbb{D}'_l\{\boldsymbol{H}_Q^{-1}\mathbf{g'}_s(t), r\}$$

$$\mathbb{F}\{\mathbf{G'}(t), r\} = \sum_s^S \mathbb{R}\{\mathbf{g'}_s(t), r\}$$

In this work, $\boldsymbol{H}_N$ denotes a Hadamard matrix of rank N, and the following notation is used to reference individual elements of the matrix:

$$\boldsymbol{H}_N = [h_{ij}^{(N)}], \ \boldsymbol{H}_N^{-1} = [\hat{h}_{ij}^{(N)}]$$

To review, the FORCES dataset of N encoded transmissions is split into S groups of Q transmissions. The transmit element i and transmit event e are reindexed to reflect this grouping:

$$\begin{aligned} i &= v + Q(l-1) \\ e &= q + Q(s-1) \end{aligned} \tag{I.1}$$

Where l and s are the transmit element and event group numbers, while v and q are the element's and event's indices within their group. In this analysis, (vl) is written in place of $(v + Q(l-1))$ and (qs) is written in place of $(q + Q(s-1))$ for space.

To align with this grouping, the dataset $\mathbf{G}(t)$ is reordered:

$$\begin{gathered} \mathbf{G}(t) \in \mathbb{R}^{N\times 1} \rightarrow \mathbf{G'}(t) \in \mathbb{R}^{Q\times S} \\ \mathbf{g'}_s(t) := \text{col}_s\mathbf{G'}(t) \ \ (s = 1, \dots, S) \\ g'_{q,s}(t) = \textstyle\sum_{l=1}^{S}\sum_{v=1}^{Q} h_{(qs),(vl)}^{(N)} s_{(vl)}(t) \end{gathered}$$

Now $\mathbf{g'}_s(t)$represents the s-th group of Q transmit events.

Thanks to its recursive Kronecker product definition, the Hadamard matrix $\boldsymbol{H}_N$ can be linearly separated into two smaller Hadamard matrices $\boldsymbol{H}_S$ and $\boldsymbol{H}_Q$ provided N, S, and Q are all powers of 2:

$$\begin{gathered} \boldsymbol{H}_N = \boldsymbol{H}_S \otimes \boldsymbol{H}_Q \\ h_{ie}^{(N)} = h_{(vl),(qs)}^{(N)} = h_{s,l}^{(S)} h_{q,v}^{(Q)} \\ \boldsymbol{H}_N = [h_{ij}^{(N)}] \end{gathered} \tag{I.2}$$

Similarly, for its inverse:

$$\begin{gathered} \boldsymbol{H}_N^{-1} = \boldsymbol{H}_S^{-1} \otimes \boldsymbol{H}_Q^{-1} \\ \hat{h}_{ei}^{(N)} = \hat{h}_{(qs),(vl)}^{(N)} = \hat{h}_{l,s}^{(S)} \hat{h}_{v,q}^{(Q)} \end{gathered} \tag{I.3}$$

Next, the reindexing and Kronecker definitions are applied to the FORCES reconstruction equation; the matrix multiplication decode step is kept within curly braces:

$$\begin{aligned} \mathbb{F}\{\mathbf{G}(t), r\} &= \mathbb{D}\{\boldsymbol{H}_N^{-1}\mathbf{G}(t), r\} \\ \text{Expand} &\Rightarrow \sum_i^N \{\sum_e^N \hat{h}_{ie}^{(N)} g_e\}(\tau_i(r)) \\ \text{Reindex} &\Rightarrow \sum_l^S \sum_v^Q \{\sum_s^S \sum_q^Q \hat{h}_{(vl),(qs)}^{(N)} g'_{q,s}\}(\tau_{(vl)}(r)) \\ \text{Split } \boldsymbol{H}_N^{-1} &\Rightarrow \sum_l^S \sum_v^Q \{\sum_s^S \sum_q^Q \hat{h}_{l,s}^{(S)} \hat{h}_{v,q}^{(Q)} g'_{q,s}\}(\tau_{(vl)}(r)) \\ \text{Reorder} &\Rightarrow \sum_s^S [\sum_l^S \hat{h}_{l,s}^{(S)} \sum_v^Q \{\sum_q^Q \hat{h}_{v,q}^{(Q)} g'_{q,s}\}(\tau_{(vl)}(r))] \\ \mathbb{F}\{\mathbf{G'}(t), r\} &= \sum_s^S [\sum_l^S \hat{h}_{l,s}^{(S)} \sum_v^Q \{\boldsymbol{H}_Q^{-1}\mathbf{g'}_s(t)\}(\tau_{(vl)}(r))] \end{aligned} \tag{I.4}$$

The matrix multiplication in curly braces is now a partial decoding of a single transmit group, and is defined as a **partially decoded dataset $\mathbf{D}(t)$**:

$$\begin{aligned} \mathbf{D}(t) &\in \mathbb{R}^{Q\times S} \\ \mathbf{d}_s(t) &= \boldsymbol{H}_Q^{-1}\mathbf{g'}_s(t) \\ d_{v,s}(t) &= \sum_{q=1}^{Q} \hat{h}_{v,q}^{(Q)} g'_{q,s}(t) \end{aligned} \tag{I.5}$$

As discussed in section II-B.2, the DAS operator associates each signal with a unique transmit element for beamforming, but, in equation I.4, DAS must be applied to Q partially decoded signals from an array with N transmit elements. Like the events, the N transmit elements are also divided into S groups of Q. Thus, a modified DAS operator is defined that associates an event group of Q signals with the l'th group of elements:

$$\mathbb{D}'_l\{\mathbf{d}_s(t), r\} = \sum_{v=1}^{Q} d_{v,s}(\tau_{v+Q(l-1)}(r)) \tag{I.6}$$

Note that while s denotes the event group this data is taken from, l is used to determine the transmit location for the delays.

Applying this new DAS operator to equation I.4 produces the READI reconstruction operator (eq. 12):

$$\mathbb{F}\{\mathbf{G'}(t), r\} = \sum_s^S [\sum_l^S \hat{h}_{l,s}^{(S)} \mathbb{D}'_l\{\boldsymbol{H}_Q^{-1}\mathbf{g'}_s(t), r\}]$$

$$\mathbb{R}\{\mathbf{g'}_s(t), r\} = \sum_l^S \hat{h}_{l,s}^{(S)} \mathbb{D}'_l\{\boldsymbol{H}_Q^{-1}\mathbf{g'}_s(t), r\}$$

$$\mathbb{F}\{\mathbf{G'}(t), r\} = \sum_s^S \mathbb{R}\{\mathbf{g'}_s(t), r\}$$

To form a single READI low-resolution image from $\mathbf{g'}_s(t)$, the modified DAS operator is called S times, once for each element group l. Each DAS iteration produces a sub-image, and the READI image is formed as the sum of these sub-images weighted by the s'th row of $\boldsymbol{H}_S^{-1}$.

Appendix II analyzes the partially decoded dataset $\mathbf{d}_s(t)$ to understand how it differs from a standard decoded FORCES dataset and explains the presence of $\hat{h}^{(S)}_{l,s}$ in the reconstruction operator.

Appendix II
READI Reconstruction Operator Analysis

To understand how the READI reconstruction operator recovers the original FORCES image we must analyze what happens when we attempt to beamform a group from the partially decoded dataset $\mathbf{d}_s(t)$ using standard delay-and-sum. First, $\mathbf{d}_s(t)$ must be defined in relation to the multistatic dataset $\mathbf{S}(t)$. Substituting the definition of $g'_{q,s}(t)$ (eq. 10) into equation I.5:

$$\begin{aligned}
d_{v,s}(t) &= \sum_{q=1}^{Q} \hat{h}^{(Q)}_{v,q}\, g'_{q,s}(t) \\
\text{Expand } g'_{q,s}(t) &\Rightarrow \sum_{q}^{Q} \hat{h}^{(Q)}_{v,q} \sum_{l=1}^{S} \sum_{v'=1}^{Q} h^{(N)}_{(qs),(v'l)} s_{(v'l)}(t) \\
\text{Split } \boldsymbol{H}_N &\Rightarrow \sum_{q}^{Q} \hat{h}^{(Q)}_{v,q} \sum_{l}^{S} \sum_{v'}^{Q} h^{(S)}_{s,l} h^{(Q)}_{q,v'} s_{(v'l)}(t) \\
\text{Reorder} &\Rightarrow \sum_{l}^{S} \sum_{v'}^{Q} h^{(S)}_{s,l} s_{(v'l)}(t) \sum_{q}^{Q} \hat{h}^{(Q)}_{v,q} h^{(Q)}_{q,v'} \\
\boldsymbol{H}_Q \boldsymbol{H}_Q^{-1} = \boldsymbol{I}_Q &\Rightarrow \sum_{l}^{S} \sum_{v'}^{Q} h^{(S)}_{s,l} s_{(v'l)}(t) \delta(v, v') \\
d_{v,s}(t) &= \sum_{l}^{S} h^{(S)}_{s,l} s_{v+Q(l-1)}(t) \qquad \text{(II.1)}
\end{aligned}$$

Thus, the v'th signal in the partially decoded dataset $\mathbf{d}_s(t)$ is a linear combination of the contributions from the v'th transmit element of every group. This is very visible in Figure 2C, where with four groups of two events ($N = 8$ $S = 4$ $Q = 2$) the partially decoded apertures contain $S = 4$ signals apiece. In general, the number of terms mixed in each partially decoded signal $d_{v,s}(t)$ is equal to the number of groups S, and their coefficients are given by the s-th row of $\boldsymbol{H}_S$.

We will apply this definition to the case of two groups of two transmit events each ($N = 4$ $S = 2$ $Q = 2$):

$$\boldsymbol{H}_S = \begin{bmatrix} 1 & 1 \\ 1 & -1 \end{bmatrix}$$

$$\mathbf{d}_1(t) = \begin{bmatrix} s_1(t) + s_3(t) \\ s_2(t) + s_4(t) \end{bmatrix} \quad \mathbf{d}_2(t) = \begin{bmatrix} s_1(t) - s_3(t) \\ s_2(t) - s_4(t) \end{bmatrix}$$

Both partially decoded groups contain all four multistatic signals, mixed according to the group's row in the $\boldsymbol{H}_S$ ($\boldsymbol{H}_2$) matrix. Attempting to directly beamform these groups using the DAS operator (equation 5) would produce images with significant artifacts. Per equation 6, the correct reconstruction of $\mathbf{G}(t)$ for an $N = 4$ FORCES sequence is:

$$\mathbb{F}\{\mathbf{G}(t), r\} = s_1(\tau_1) + s_2(\tau_2) + s_3(\tau_3) + s_4(\tau_4)$$

Where the contribution from each transmitting element $s_i(t)$ is delayed according to that element's position τ_i, bringing the point into focus. However, attempting standard DAS beamforming on $\mathbf{d}_1(t)$ yields:

$$\mathbb{D}\{\mathbf{d}_1(t), r\} = s_1(\tau_1) + s_2(\tau_2) + s_3(\tau_1) + s_4(\tau_2)$$

The elements of group one (s_1 and s_2) are beamformed with correct delays, whereas the second group (s_3 and s_4) is incorrectly delayed and out of focus. To obtain all four in-focus signals $\mathbf{d}_1(t)$ must be beamformed twice with the modified DAS operator (I.6), once for each element group:

$$\mathbb{D}'_2\{\mathbf{d}_1(t), r\} = s_1(\tau_3) + s_2(\tau_4) + s_3(\tau_3) + s_4(\tau_4)$$

$$\begin{aligned}
\mathbb{R}\{\mathbf{g'}_1(t), r\} = \mathbb{D}'_1\{\mathbf{d}_1(t), r\} + \mathbb{D}'_2\{\mathbf{d}_1(t), r\} = \\
s_1(\tau_1) + s_2(\tau_2) + s_3(\tau_3) + s_4(\tau_4) \\
+ s_1(\tau_3) + s_2(\tau_4) + s_3(\tau_1) + s_4(\tau_2) \qquad \text{(II.2)}
\end{aligned}$$

This produces an image that contains all in-focus data present in the full FORCES image, but with significant artifacts from extra cross terms. We refer to this image as a **READI low-resolution image**, while the individual outputs of the $\mathbb{D}'_l\{\mathbf{d}_s(t), r\}$ operators are referred to as **READI sub-images**.

A second issue arises when we attempt to beamform $\mathbf{d}_2(t)$. As its components are weighted by the second row of $\boldsymbol{H}_2$ ($[1, -1]$) all terms from the second group of elements (3 and 4) are negative. To counteract this, we subtract the second sub-image from the first when forming the READI low-resolution image:

$$\begin{aligned}
\mathbb{R}\{\mathbf{g'}_2(t), r\} = \mathbb{D}'_1\{\mathbf{d}_2(t), r\} - \mathbb{D}'_2\{\mathbf{d}_2(t), r\} = \\
s_1(\tau_1) + s_2(\tau_2) + s_3(\tau_3) + s_4(\tau_4) \\
- s_1(\tau_3) - s_2(\tau_4) - s_3(\tau_1) - s_4(\tau_2) \qquad \text{(II.3)}
\end{aligned}$$

In general, when forming the READI low-resolution image for the s-th group, the sub-images are weighted according to the s-th column of $\boldsymbol{H}_S^{-1}$, which ensures that all in-focus terms have the same sign. Adding this weighting ($\hat{h}^{(S)}_{l,s}$) completes the definition of the READI reconstruction operator:

$$\mathbb{R}\{\mathbf{g'}_s(t), r\} = \sum_{l}^{S} \hat{h}^{(S)}_{l,s} \mathbb{D}'_l\{\boldsymbol{H}_Q^{-1} \mathbf{g'}_s(t), r\}$$

Both READI low-resolution images now contain all in-focus terms, along with a set of out-of-focus cross-terms that degrade image quality. When these two low-resolution images are summed, these cross-terms cancel out, leaving only the original FORCES image:

$$\begin{aligned}
\mathbb{F}\{\mathbf{G'}(t), r\} &= \mathbb{R}\{\mathbf{g'}_1(t), r\} + \mathbb{R}\{\mathbf{g'}_2(t), r\} \\
&= s_1(\tau_1) + s_2(\tau_2) + s_3(\tau_3) + s_4(\tau_4)
\end{aligned}$$

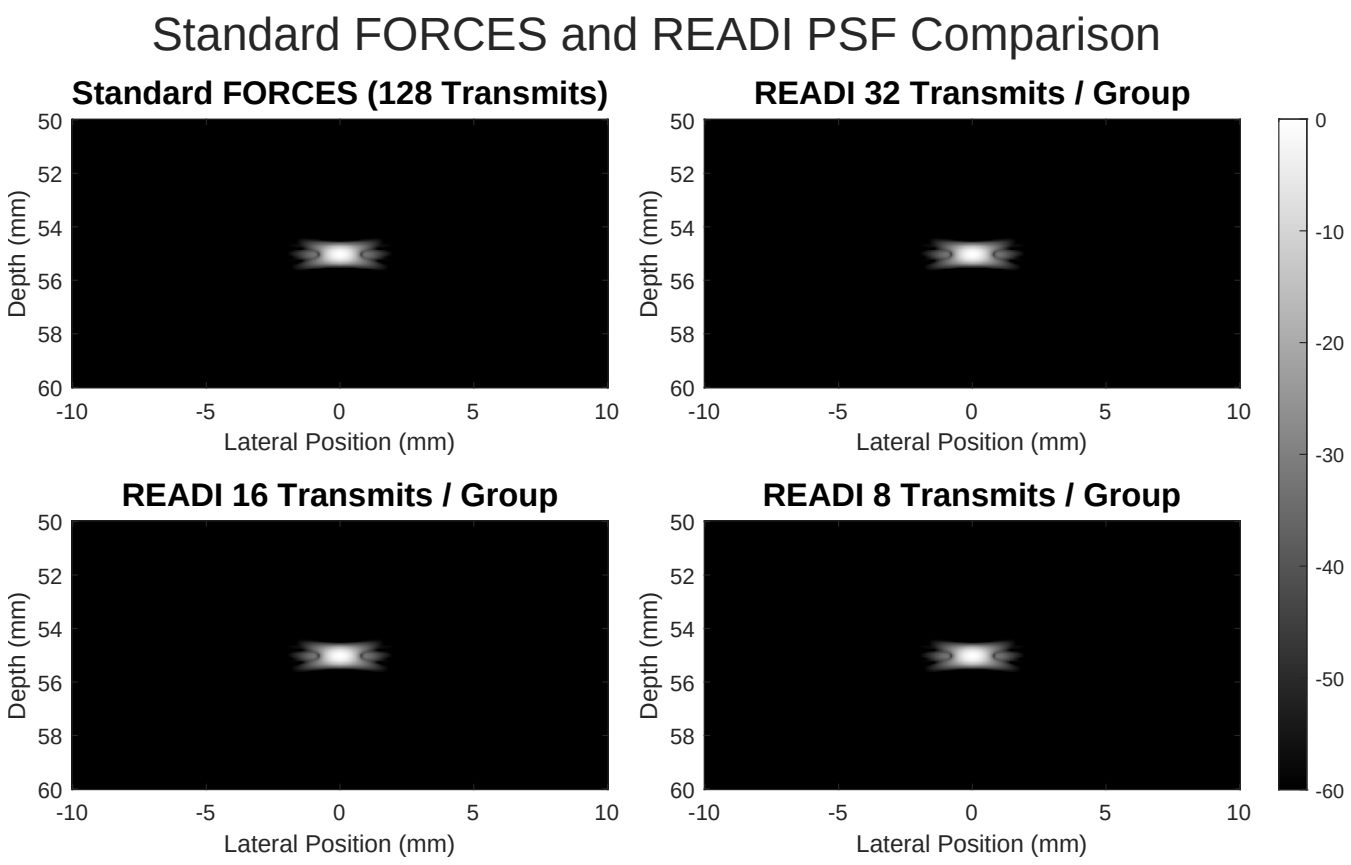

Fig. A.1. Point Spread Function simulation in Field II, comparing standard FORCES beamforming with READI beamforming using groups of 32, 16, and 8 transmits. All images are identical within numerical precision.

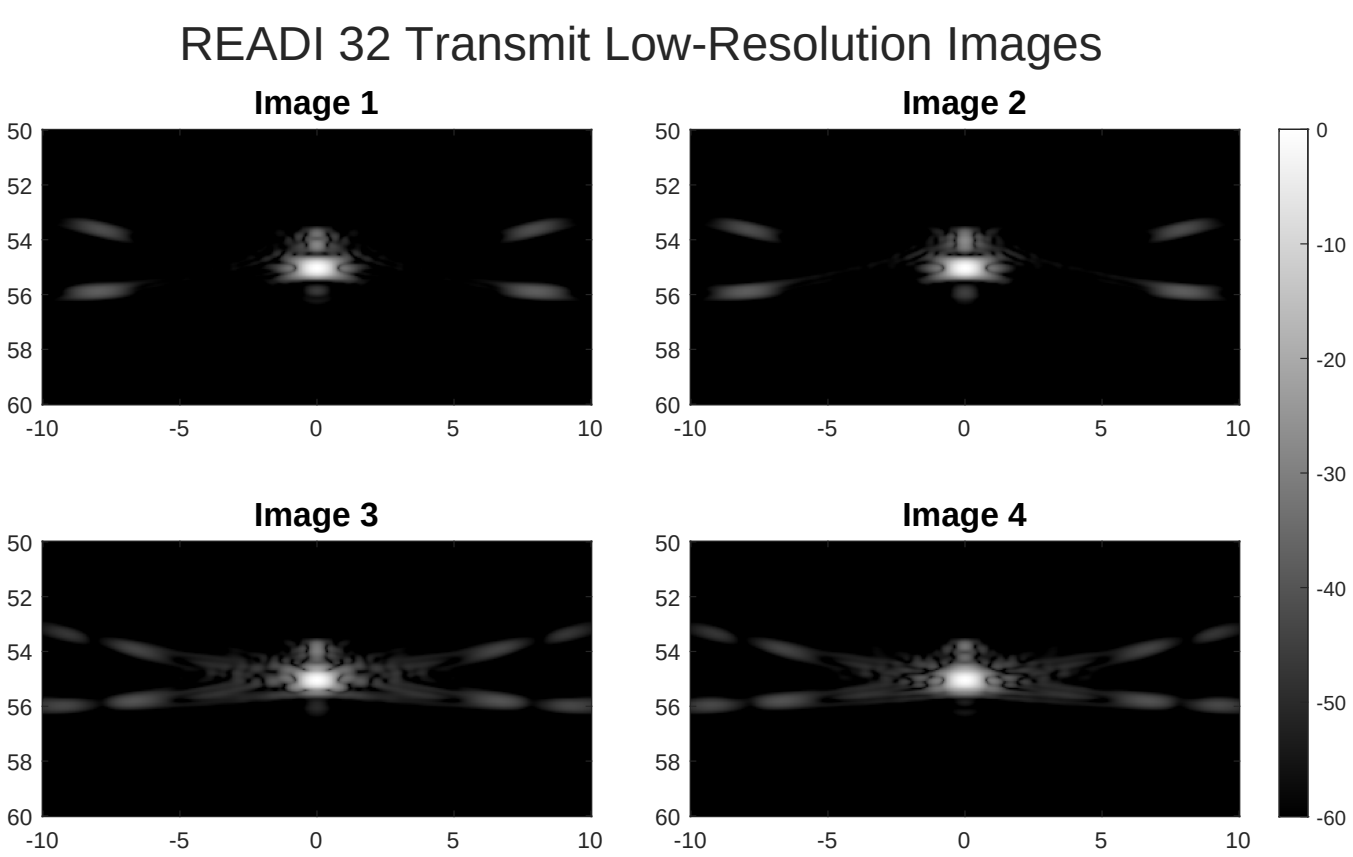

Fig. A.2. READI low-resolution point spread functions formed from 32 transmits apiece. Coherently summing these four images produces the top right image in Figure A.1.

Appendix III
Additional Results

A. Point Spread Function Simulations

Coherence-factor weighting was disabled in these simulations to preserve linearity.

To validate the READI beamforming process, a 128-transmit FORCES PSF was simulated in Field II. The recorded data was beamformed four times, once using the standard FORCES beamformer and three times using READI, with group sizes (Q) of 32, 16, and 8 transmits. Figure A.1 displays the beamformed images. In all cases, the READI beamformer successfully reproduces the static FORCES image, with average per-pixel errors on the order of 10^{-3} dB at a dynamic range of 100 dB. This error is attributable to 32-bit floating-point precision limitations and is negligible compared to the noise floor of real acquisitions.

Figure A.2 displays the four low-resolution READI PSFs formed with $Q = 32$. Each PSF is centred at the same position but has its own set of unique artifacts. These READI artifacts are generated by the cross-terms in the partially decoded dataset (described in Appendix II equations II.2 and II.3). Each group has a distinct set of artifacts due to the different signs of its cross-terms; when all images are summed, these artifacts cancel out.

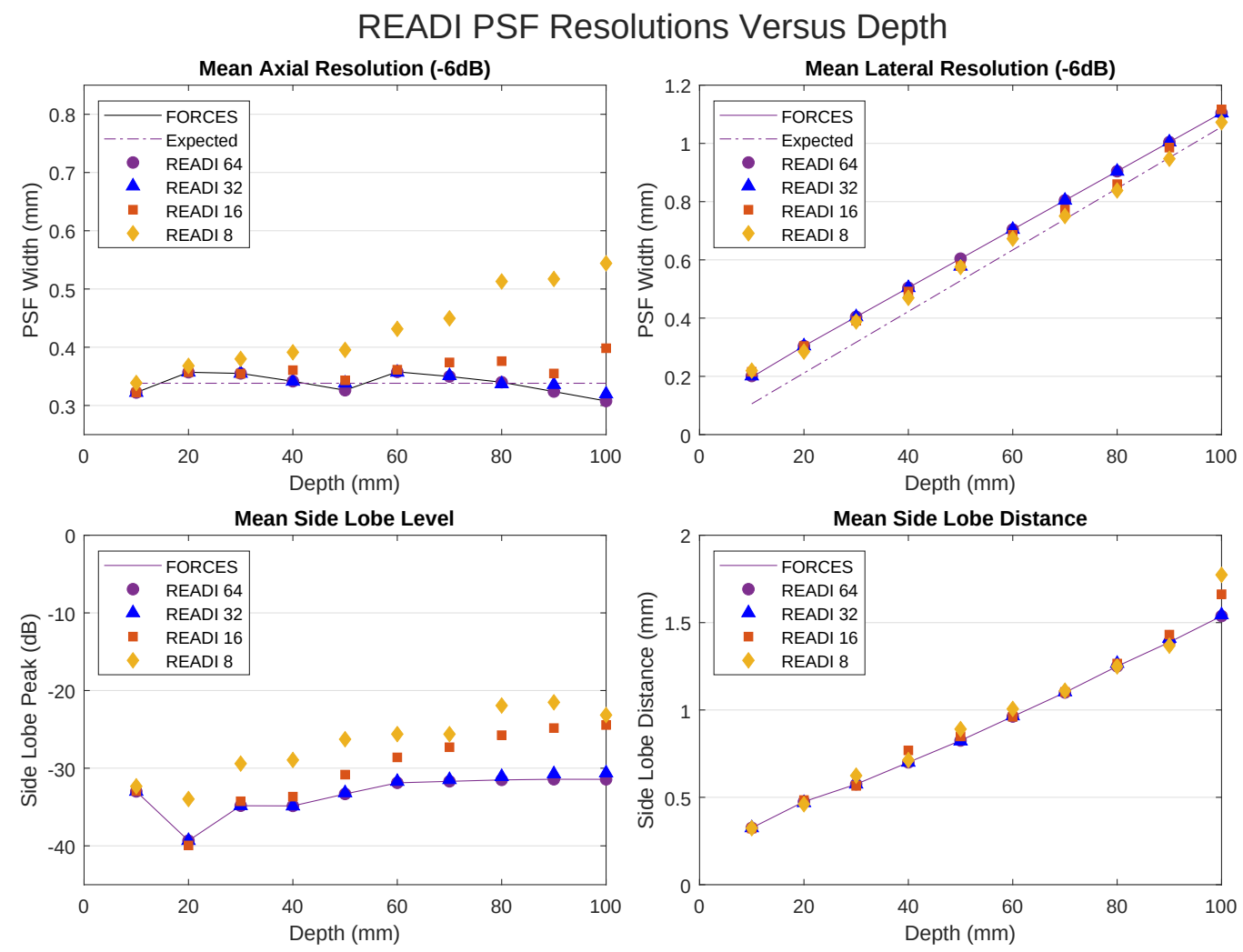

Fig. A.3. Field II PSF resolution measurements for FORCES and READI with different group sizes. All READI results are mean values from the low-resolution images.

Array Type	128x128 TOBE
Transmit Frequency	4.3 MHz
Element Pitch	0.25 mm
Transmit Waveform	2-cycle sinusoid
Sampling Frequency	50 MHz
Speed of Sound	1540 m/s
Elevational Focal Depth	55 mm
Transmit Apodization Pattern	Hanning

TABLE A.I
Field II PSF Simulation Array Parameters

To analyze the resolution characteristics, the stationary FORCES PSF was simulated at various depths and beamformed with multiple READI group sizes (64, 32, 16, and 8 transmits). The axial and lateral resolution was measured at each depth, along with sidelobe position and height. The results are averaged across all READI images for each group size. Results are presented in Figure A.3. All group counts have similar lateral resolution, closely following the FORCES image and the expected resolution of $\lambda F_{\#}$ for a wavelength of 330 μm and a 32 mm wide array. Axially, all cases stay close to the expected value of λ for a fully focused 2-cycle pulse; however, the reduced transmit focusing begins to have noticeable effects on READI 8 at lower depths. Since each READI 8 image only contains 8 transmissions, this is an understandable loss of quality. We also observe that the number of READI groups has no effect on the nearest sidelobe position (defined as the first lateral peak in the PSF off-center); however, as the READI group count increases, sidelobe heights can also increase. Table A.I lists all relevant simulation parameters for this work and the simulation results presented in the main text. All simulation code is available upon request.

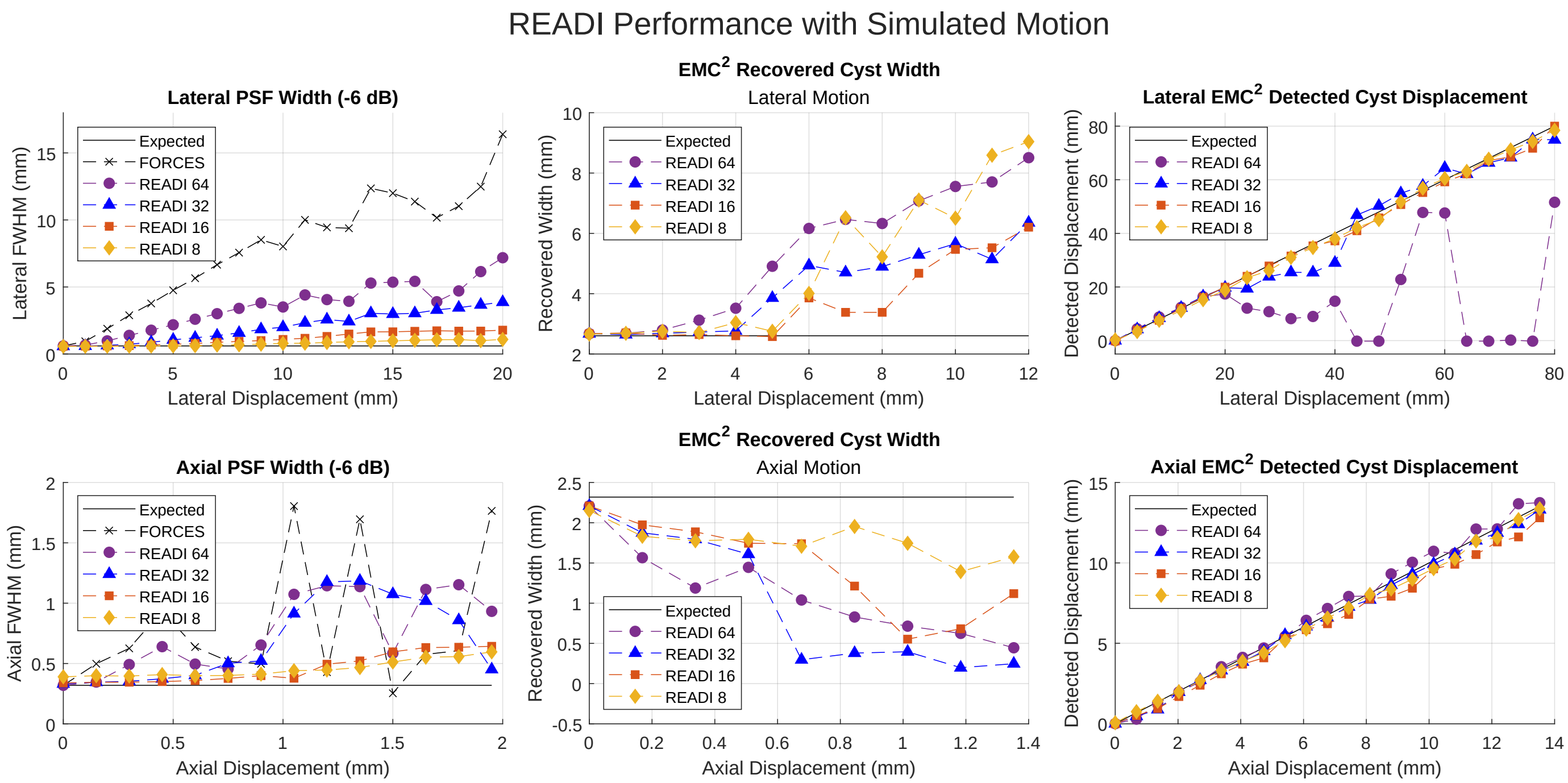

Fig. A.4. Field II READI performance with axial and lateral motion. Displacement values state the total amount of motion over the 128 transmit FORCES sequence.

Number of Scatterers	280902
Lateral Scatterer Range	-45 to 45 mm
Elevational Scatterer Range	-1 to 1 mm
Axial Scatterer Range	40 to 60 mm
Cyst Diameter	2 mm
Cyst Shape	Spherical
Cyst Scatterer Strength	15x Background Strength

TABLE A.II
FIELD II CYST SIMULATION SCATTERER PARAMETERS

B. Motion Simulation

Two simulations were performed to analyze axial and lateral performance, using the same parameters as in the resolution analysis above. First, a PSF is simulated moving at constant axial and lateral rates, and its resolution in the relevant direction is measured. Then, a 2 mm-diameter spherical hyperechoic cyst is moved against a uniform background of speckle. Its motion was estimated with NCC, and EMC^2 attempted to reconstruct the cyst. 30 dB of white noise was added to the simulated RF data to more closely approximate real-world conditions. All test cases are expressed as displacements (the distance the point moves over the 128 FORCES transmissions). These results are collected in Figure A.4. The simulation settings for the PSFs and the array are the same as provided in Table A.I. Table A.II describes the scatterer parameters for the cyst simulation.

Starting with the PSF measurements on the left of Figure A.4, we observe that the measured PSF width scales proportionally with the amount of lateral motion. As the sequence is divided into READI groups, the slope of this curve is also divided by the number of groups. Axially, this trend is less stable. In the FORCES case, we see an abrupt spike in PSF width as soon as motion begins. With READI 2, this spike is shifted further out, and with smaller group sizes, it smooths into a gradual increase. In both cases, the measured resolution remains significantly more stable at higher READI group counts.

For the cyst motion simulation (center and right), we observe that EMC^2 fails to recover the correctly sized cyst at 10 mm of lateral displacement and 1 mm of axial displacement (over 128 transmissions), yet the measured motion fields remain accurate far past these cutoffs. READI accurately measures cyst motion with up to 80 mm of lateral displacement and up to 14 mm of axial displacement. At a PRF of 1 kHz, these correspond with speeds of 62.5 cm/s and 11.0 cm/s, respectively; at 3.5 kHz, they scale to 187.5 cm/s and 48.5 cm/s. These extremely high speeds indicate that, even after EMC^2 fails to reconstruct the static image, READI is still capable of delivering accurate motion estimates. All simulation code is available upon request.

C. Heart Phantom Results

This section contains additional results from the heart phantom experiment. Figure A.5 shows all 8 READI low-resolution images from the experiment, which are the inputs into EMC^2 and produce the results in Figures 8 and 9. Figure A.6 shows all 7 motion fields detected by EMC^2 in this experiment, along with an average of their magnitudes.

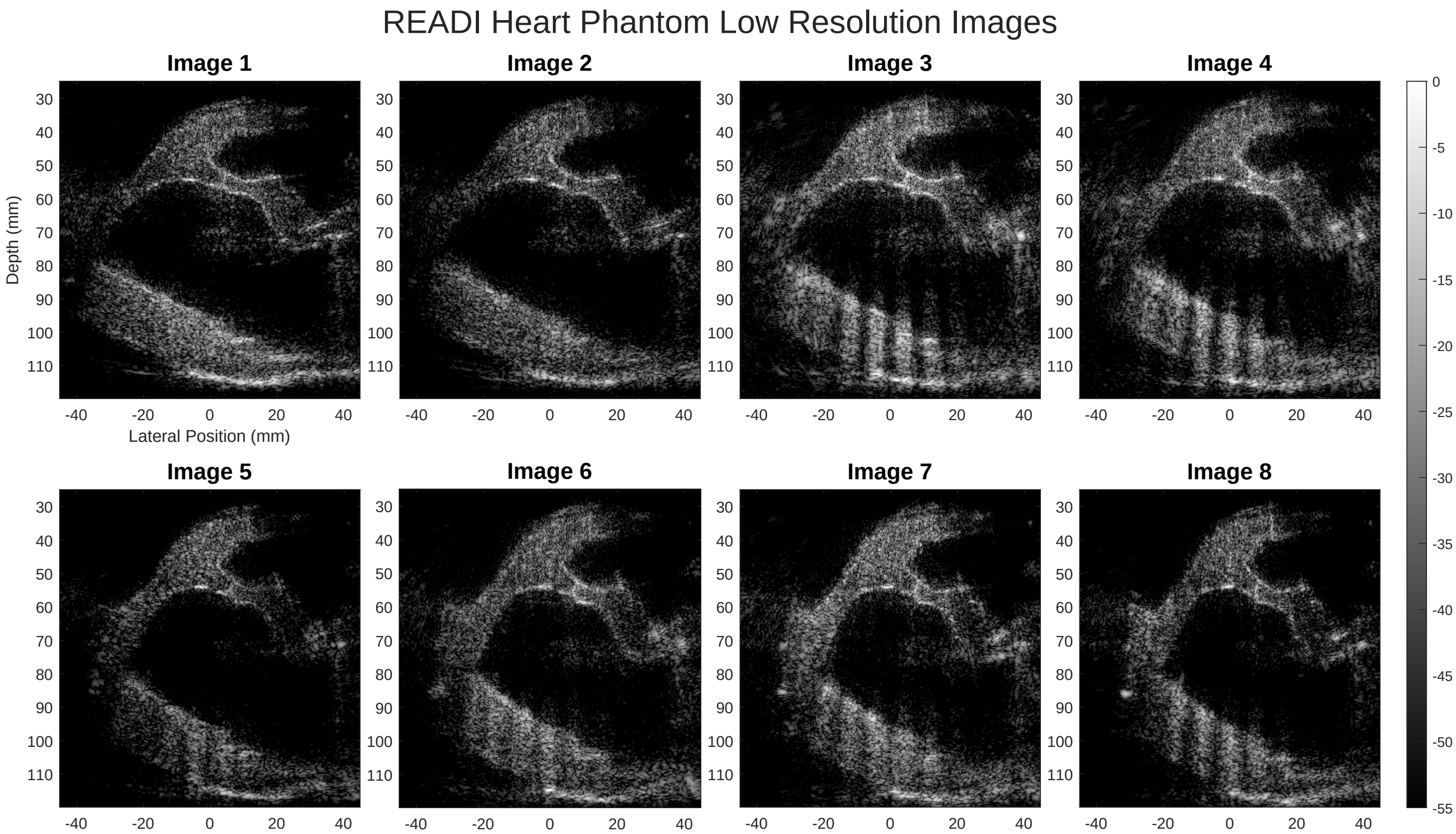

Fig. A.5. The 8 READI low-resolution images from the heart phantom experiment. These images in the input into EMC^2 and produce the results in Figures 8 and 9

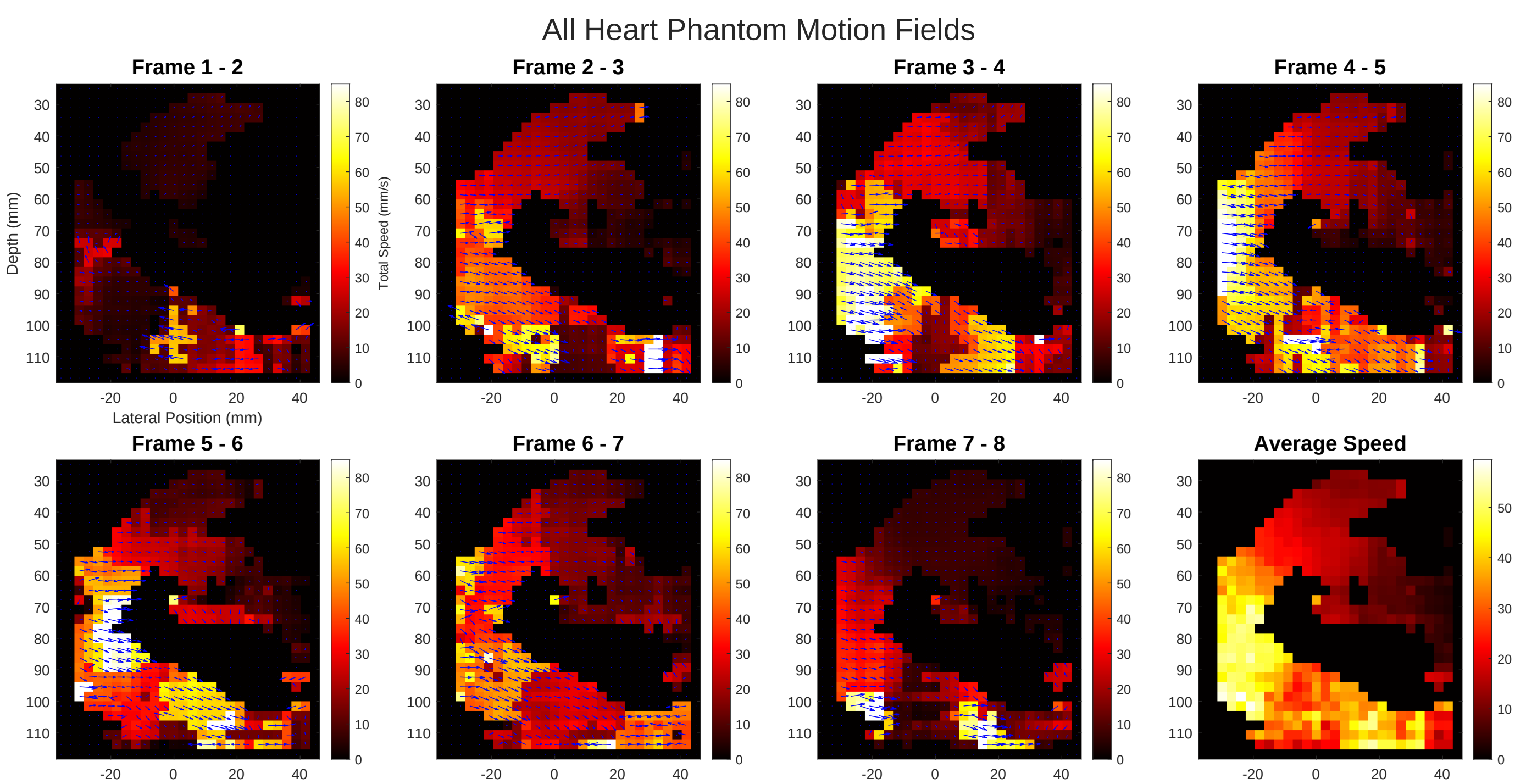

Fig. A.6. Collection of all 7 motion fields detected by EMC^2 in the heart phantom experiment. Each field has been downsampled by a factor of 2 for visualization purposes. Background colour indicates the absolute value of the field in mm/s. The final image is the average of the 7 fields' magnitudes and has a lower colour scale ceiling.
